# Natural products and extracellular vesicle-associated miRNA networks in perianal infectious wound healing: a critical ethnopharmacological appraisal

**DOI:** 10.3389/fphar.2026.1917327

**Published:** 2026-10-29

**Authors:** Wei Yang, Ruizhen Zhang, Jiamin Hang, Yujie Su, Yulong Ma, Qiang Song, Cong Wang, Xi Zhang

**Affiliations:** 1 Xi’an Hospital of Traditional Chinese Medicine, Xi’an, Shaanxi, China; 2 Southern Medical University, Guangzhou, Guangdong, China

**Keywords:** biofilm, extracellular vesicles, microRNA, natural products, perianal infectious wounds, topical delivery, wound healing

## Abstract

Perianal infectious wounds form a contaminated, moist, mechanically disturbed repair niche that differs from standard cutaneous wounds. This critical narrative review evaluates whether natural-product pharmacology and extracellular vesicle (EV)-associated miRNA biology can be linked credibly in this setting. A structured PubMed search updated through 29 September 2026, supplemented by Google Scholar citation tracking, identified direct human perianal studies, anatomically relevant and infection-relevant animal studies, and mechanistically relevant *in vitro* and *in silico* evidence. Core studies were appraised on two separate axes: clinical/model proximity and methodological credibility, including material identity and taxonomic validity, chemical characterization, exposure, comparators and controls, model fidelity, EV characterization, causal miRNA evidence, and assay-interference risk. Direct human ethnopharmacological evidence now includes several randomized postoperative studies, although blinding, material standardization, comparator quality, endpoint selection, and mechanistic depth remain heterogeneous. Early-phase mesenchymal stromal cell EV studies provide perianal feasibility and fistula-closure signals without controlled comparative efficacy. A 2025 Wugu Qilin study provides direct human treatment-associated evidence from wound-fluid vesicle-enriched miRNA profiling involving miR-93-3p/EIF4EBP1, but it did not demonstrate EV-mediated transfer to recipient cells. Astragaloside IV-conditioned endothelial progenitor-cell EV studies provide stronger nonperianal cargo-level evidence, whereas curcumin-loaded macrophage EVs primarily support vesicle-assisted delivery. No study establishes the full causal sequence from chemically defined natural-product exposure through rigorously characterized EV cargo alteration and transfer of a defined miRNA to a necessary recipient-cell effect and improved healing in an anatomically faithful perianal model. Direct clinical and translational evidence is therefore present, but the natural product-EV-miRNA mechanism remains unproven; the most immediate needs are material standardization, clinically derived wound-fluid EV profiling, mixed-biofilm models, causal cargo perturbation, assay-interference controls, and stage-adapted local delivery.

## 1 Introduction

Perianal infectious wounds arise most commonly after anorectal abscess, fistula-in-ano, or surgery for anorectal infection and can remain painful, exudative, and slow to close. Their biological context differs from a conventional dry cutaneous wound: the wound bed is repeatedly exposed to stool, moisture, friction, mixed aerobic and anaerobic microorganisms, and irregular cavities or fistula tracts. Population studies confirm a clinically meaningful burden of fistula disease and recurrent care, but the translational problem addressed here is not fistula prevalence itself; it is the persistence of a contaminated repair niche after drainage or surgery (Sarveazad et al., 2022; Sanchez-Haro et al., 2023; Gaertner et al., 2022).

Source control remains indispensable. Drainage, anatomical assessment, debridement when required, antimicrobial treatment when indicated, and sphincter-preserving surgery provide the basis for healing (Gaertner et al., 2022). However, technically adequate treatment does not necessarily normalize the wound microenvironment. Anal fistula-associated microbiota can reflect both intestinal and skin sources, and prolonged fistulas may contain bacteria with substantial biofilm-forming capacity (Jaiswal et al., 2021; Yang et al., 2024). These features make persistent microbial stimulation, inflammatory dysregulation, impaired angiogenesis, fibroblast dysfunction, and unstable epithelial repair more relevant to this review than a general description of wound-healing phases.

Within contemporary Chinese medicine practice, external washing or sitz-bath treatment, ointment or gauze application, and increasingly hydrogel-based local delivery are used as adjuncts after anal-fistula or perianal-abscess surgery. Published human studies range from earlier topical oil and decoction studies (Li et al., 2009; Deng et al., 2022) to recent randomized trials (Wang J. et al., 2026; Zhao et al., 2026) and wound-fluid molecular analyses (Zhang et al., 2025). Their evidentiary value varies substantially because randomization, blinding, botanical or material characterization, comparator choice, and endpoint quality are inconsistent. Traditional use is therefore treated as a rationale for pharmacological investigation rather than as evidence of efficacy.

Plant-derived metabolites and botanical preparations are attractive in this setting because they may influence microbial behavior, inflammatory signaling, oxidative stress, vascular repair, stromal function, and local delivery. At the same time, extracellular vesicles (EVs) provide a plausible route through which donor-cell state can alter communication among macrophages, endothelial cells, fibroblasts, and keratinocytes. EV-associated miRNAs such as miR-146a, miR-21, miR-126, miR-132, miR-145-5p, and miR-223 have shown repair-related effects in experimental wounds, while miR-24-3p and persistent miR-155 activity illustrate that vesicle cargo can also be maladaptive. The key uncertainty is whether these findings can be transferred to the contaminated perianal environment and whether plant-derived interventions truly modulate the relevant EV-miRNA pathways rather than merely sharing downstream signaling readouts.

This question is distinct from the scope of recent reviews. Broad syntheses already cover natural products in wound healing (Raza et al., 2024), possible combinatorial use of plant-derived metabolites and extracellular vesicles for tissue repair (Koyama et al., 2024), plant-derived extracellular vesicles in diabetic wounds (Xu et al., 2025), and the preclinical efficacy of plant-derived EVs in diabetic wound models (Wu et al., 2026). Perianal and intestinal EV therapy are also no longer unreviewed fields: recent publications include a focused review of stem-cell/EV therapies for perianal fistulizing Crohn’s disease (Bhatnagar et al., 2025), a systematic review/single-arm meta-analysis of MSC-derived exosomes for complex perianal fistula (Cheng et al., 2025), a 2026 review of regenerative therapies for perianal fistulizing Crohn’s disease that includes EV approaches (Takata et al., 2026), and a September 2026 review of EV-based therapies across IBD (Lu et al., 2026). Against this background, the present review focuses on a specific ethnopharmacological question: which natural-product and EV-miRNA findings are sufficiently characterized and model-relevant to inform perianal infectious wound repair, which claims remain vulnerable to poor material characterization, assay interference, indirect anatomy, or weak causal design, and what evidence is still missing at the intersection of these fields?

Direct human perianal evidence is considered separately from anatomically direct animal models, infection-relevant wound models, other wound models, cell studies, and *in silico* analyses. Within each tier, emphasis is placed on what the original study design can and cannot support rather than on the number of positive reports. Cross-model extrapolation is labelled explicitly, and clinically or mechanistically informative studies are examined in greater detail when their design materially changes the interpretation of the evidence. This structure also allows practical validation priorities to be derived from specific gaps in material characterization, model fidelity, EV methodology, causal miRNA testing, and local delivery after adequate source control.

## 2 Literature search, study selection, and evidence appraisal

### 2.1 Updated literature search strategy

A structured literature search was conducted from database inception through 29 September 2026. PubMed was the primary bibliographic database because it permits transparent and reproducible indexed searching of biomedical and pharmacological evidence. Google Scholar was used for forward and backward citation tracking and to locate potentially relevant non-PubMed-indexed reports in the anorectal and ethnopharmacological literature; it was not used as the sole basis for evidence inclusion or quantitative record counting. No language restriction was applied at the search stage. Studies were retained only when the model, intervention, and principal findings could be identified with sufficient confidence, and incompletely reported methodological details were recorded as limitations.

Because the literature spans a narrow direct intersection and a much larger body of mechanistically relevant bridging evidence, the PubMed search was organized into five structured concept blocks defined for this review rather than one overly restrictive query. The following Title/Abstract searches were used:

Search 1, perianal/anorectal wounds and natural products:

((perianal [Title/Abstract] OR “anal fistula” [Title/Abstract] OR “fistula-in-ano” [Title/Abstract] OR anorectal [Title/Abstract]) AND (“wound healing” [Title/Abstract] OR wound*[Title/Abstract]) AND ((“natural product” [Title/Abstract] OR “natural products” [Title/Abstract]) OR phytochemical*[Title/Abstract] OR herbal [Title/Abstract] OR botanical [Title/Abstract] OR “traditional Chinese medicine” [Title/Abstract] OR metabolite*[Title/Abstract] OR extract*[Title/Abstract])).

Search 2, perianal/anorectal wounds and extracellular-vesicle/miRNA biology: ((perianal [Title/Abstract] OR “anal fistula” [Title/Abstract] OR “fistula-in-ano” [Title/Abstract] OR anorectal [Title/Abstract]) AND (exosome*[Title/Abstract] OR (“extracellular vesicle” [Title/Abstract] OR “extracellular vesicles” [Title/Abstract]) OR miRNA*[Title/Abstract] OR microRNA*[Title/Abstract])).

Search 3, wound models at the natural-product–EV/miRNA intersection: (((“infected wound” [Title/Abstract] OR “infected wounds” [Title/Abstract]) OR (“chronic wound” [Title/Abstract] OR “chronic wounds” [Title/Abstract]) OR (“diabetic wound” [Title/Abstract] OR “diabetic wounds” [Title/Abstract]) OR (“cutaneous wound” [Title/Abstract] OR “cutaneous wounds” [Title/Abstract]) OR (“pressure ulcer” [Title/Abstract] OR “pressure ulcers” [Title/Abstract]) OR “wound healing” [Title/Abstract]) AND ((“natural product” [Title/Abstract] OR “natural products” [Title/Abstract]) OR phytochemical*[Title/Abstract] OR herbal [Title/Abstract] OR botanical [Title/Abstract] OR metabolite*[Title/Abstract] OR extract*[Title/Abstract]) AND (exosome*[Title/Abstract] OR (“extracellular vesicle” [Title/Abstract] OR “extracellular vesicles” [Title/Abstract]) OR miRNA*[Title/Abstract] OR microRNA*[Title/Abstract])).

Search 4, representative plant-derived metabolites and wound/biofilm evidence: ((“wound healing” [Title/Abstract] OR (“infected wound” [Title/Abstract] OR “infected wounds” [Title/Abstract]) OR (“diabetic wound” [Title/Abstract] OR “diabetic wounds” [Title/Abstract]) OR biofilm*[Title/Abstract]) AND (berberine [Title/Abstract] OR baicalin [Title/Abstract] OR baicalein [Title/Abstract] OR curcumin [Title/Abstract] OR quercetin [Title/Abstract] OR resveratrol [Title/Abstract] OR “salvianolic acid B” [Title/Abstract] OR astragaloside*[Title/Abstract] OR ginsenoside*[Title/Abstract] OR asiaticoside [Title/Abstract] OR madecassoside [Title/Abstract] OR “glycyrrhizic acid” [Title/Abstract])).

Search 5, wound-relevant EV-associated miRNAs: ((“wound healing” [Title/Abstract] OR (“infected wound” [Title/Abstract] OR “infected wounds” [Title/Abstract]) OR (“diabetic wound” [Title/Abstract] OR “diabetic wounds” [Title/Abstract]) OR (“cutaneous wound” [Title/Abstract] OR “cutaneous wounds” [Title/Abstract])) AND (exosome*[Title/Abstract] OR (“extracellular vesicle” [Title/Abstract] OR “extracellular vesicles” [Title/Abstract])) AND (“miR-146a” [Title/Abstract] OR “miR-21” [Title/Abstract] OR “miR-126” [Title/Abstract] OR “miR-132” [Title/Abstract] OR “miR-145-5p” [Title/Abstract] OR “miR-223” [Title/Abstract] OR “miR-24-3p” [Title/Abstract] OR “miR-155” [Title/Abstract] OR miRNA*[Title/Abstract] OR microRNA*[Title/Abstract])).

Reference lists of eligible original studies and recent reviews were hand-searched, and forward citation tracking was performed in Google Scholar to identify additional perianal, anal-fistula, infected-wound, extracellular-vesicle, and natural-product studies. Reviews and systematic reviews were used for contextualization, evidence mapping, and citation discovery but were not treated as primary evidence for pharmacological efficacy. This was a critical narrative review rather than a systematic review or meta-analysis; the structured search was used to make study identification transparent and reduce arbitrary selection, not to claim exhaustive capture or support quantitative pooling.

Retraction, correction, and update status was checked for studies carrying principal efficacy or mechanistic claims. Retracted publications were excluded from the evidentiary synthesis, published corrections or updated versions were consulted when available, and multiple reports arising from the same research group or closely related experimental platform were not treated as independent replication without supporting evidence of independence.

### 2.2 Eligibility and study selection

Original human, animal, *ex vivo*, and *in vitro* studies were eligible when they addressed at least one of the following: (i) healing of perianal, anal-fistula, anorectal, infected, chronic, diabetic, pressure-ulcer, burn, or other mechanistically relevant wounds; (ii) extracellular-vesicle-mediated regulation of wound repair; (iii) wound-relevant miRNA cargo or causal miRNA signaling; (iv) pharmacological or delivery effects of a natural product, botanical preparation, or plant-derived metabolite relevant to inflammation, infection or biofilm, angiogenesis, fibroblast/keratinocyte function, extracellular-matrix remodeling, or epithelial repair; or (v) topical delivery systems directly relevant to the moist and contaminated perianal environment. Completed clinical studies were distinguished from trial protocols, and protocols were not interpreted as evidence of efficacy. Case reports were retained only when they added otherwise unavailable direct clinical information and were identified explicitly as case-level evidence. Network-pharmacology, docking, and other *in silico* studies were retained only for hypothesis generation or contextual target prioritization and were not used as stand-alone evidence of pharmacological activity or mechanism.

Studies were excluded when wound relevance was absent, the intervention or biological material could not be identified with sufficient confidence, the article provided only a secondary summary without original data, or the reported assay/model was too remote from the question to support the claimed wound-healing activity. When multiple reports described the same experimental platform, the report providing the most complete pharmacological, mechanistic, or translational information was prioritized.

The review question was: which natural-product, EV, and miRNA evidence is sufficiently characterized and model-relevant to inform hypotheses for perianal infectious wound repair, and which claims remain too indirect or methodologically weak for that inference? For each core original study, data extraction covered study design and model, intervention identity and source, botanical or other material authentication where relevant, dose or concentration and duration, comparator(s), chemical/material or EV characterization, principal wound-related endpoints, causal perturbation when performed, and study-specific limitations. Unreported methodological or exposure details were recorded as limitations rather than inferred from secondary sources. Evidence tables were designed around completed original studies that carried the principal clinical, translational, or mechanistic claims. Protocols, reviews, systematic reviews, meta-analyses, guidelines, and consensus documents were not mixed into completed primary-study tables; when methodologically informative, they were discussed separately in the text. Given the heterogeneity of clinical trials, animal models, cell and microbial assays, biomaterials, and *in silico* studies, no quantitative synthesis was attempted.

### 2.3 Evidence hierarchy and critical appraisal

Evidence was interpreted on two separate axes. The first axis was proximity to the clinical question: level 1, completed human studies in perianal or anorectal wounds; level 2, anatomically direct *in vivo* perianal or anal-fistula models; level 3, *in vivo* infected-wound models with relevant microbial or biofilm features but without direct perianal anatomy; level 4, other *in vivo* wound models, including diabetic, pressure-ulcer, burn, and full-thickness cutaneous wounds; level 5, *ex vivo* or cell-based mechanistic studies; and level 6, *in silico*/network-pharmacology evidence or other indirect mechanistic evidence. The second axis was methodological credibility within each level. Human studies were examined for randomization, allocation concealment, blinding, comparator quality, sample size, clinically meaningful outcomes, follow-up, and confounding; animal studies for randomization or blinding, model fidelity, dose-response, comparator design, and replication; *in vitro* studies for concentration plausibility, viability controls, orthogonal readouts, counter-screens, and causal perturbation; and EV/miRNA studies for vesicle characterization, contamination controls, cargo manipulation, target validation, and rescue. Evidence level was not equated with study quality, and no single numerical score was used to collapse these design-specific domains. Because no single validated risk-of-bias instrument is applicable across the entire heterogeneous evidence base, the critical-appraisal tables use a design-specific domain approach rather than a pooled quality score.

Evidence level reflects anatomical and clinical proximity, not etiologic equivalence. Thus, Crohn’s perianal-fistula studies can be Level 1 or Level 2 because they directly address the perianal fistula compartment, but their immune-mediated inflammatory pathogenesis differs from cryptoglandular or postoperative infectious wounds. Etiologic match, microbial context, source control, fistula geometry, and wound contamination were therefore assessed separately when judging translational relevance.

For pharmacological studies, the appraisal extracted, when reported, the identity and source of the test material, botanical authentication and taxonomic validity for plant preparations, plant part and voucher information where applicable, extraction or formulation method, chemical characterization or purity, tested concentration or dose range, minimum active concentration/dose where available, exposure duration, positive and negative controls, cytotoxicity or tolerability controls, model relevance, and replication of the principal effect. These domains were evaluated in accordance with the Four Pillars of Best Practice in Ethnopharmacology and the ConPhyMP recommendations for medicinal-plant extract characterization (Heinrich et al., 2020; Heinrich et al., 2022). Taxonomic nomenclature for identifiable plant materials was checked against the Royal Botanic Gardens, Kew Medicinal Plant Names Services (MPNS) and Plants of the World Online (POWO) (accessed 18 September 2026; Royal Botanic Gardens, Kew, 2026a; Royal Botanic GardensKew, 2026b). Accepted names are presented with botanical authorities and family, while names used in the original studies are retained when they represent synonyms or historical usage. Materials reported only by crude-drug/common name, unspecified essential oil, or another source lacking species-level identification were retained at the taxonomic level supported by the primary report, and this uncertainty was considered in the appraisal. Similarly, when an original report did not document authentication, voucher deposition, chemical fingerprinting, or exact exposure, the omission was recorded rather than reconstructed from secondary sources. Poorly characterized extracts, pharmacologically implausible exposure conditions, absent controls, or conclusions based only on screening assays were down-weighted rather than treated as equivalent to well-characterized *in vivo* or clinical evidence. For mixed-origin traditional preparations containing botanical, zoological, mineral, or other materials, the material classes were distinguished and missing provenance, authentication, or sourcing documentation was recorded as a limitation. For isolated plant-derived metabolites, original concentration units were retained for fidelity; nominal molar equivalents were added where molecular form was unambiguous and conversion was not misleading, whereas salts, mixtures, and formulation concentrations were not forcibly converted.

Extracellular-vesicle studies were additionally assessed for source definition, isolation/separation method, particle-size and morphology assessment, positive and negative markers, contamination controls, and functional evidence consistent with MISEV2023 principles (Welsh et al., 2024). Claims assigning a wound effect to a specific miRNA were considered stronger when supported by gain-of-function or loss-of-function experiments, target validation, rescue experiments, or other causal manipulations rather than association alone. Representative EV-associated miRNAs are summarized in Table 1, and design-specific appraisals of direct and mechanistic primary studies are presented in Tables 2, 3.

**TABLE 1:** Representative EV-associated miRNAs involved in wound healing regulation.

miRNA	EV source or model	Main recipient cell	Principal target or pathway	Reported wound-related effect	Relevance to perianal infectious wounds	References
miR-146a	Engineered EVs combined with a silk fibroin patch	Inflamed wound cells and macrophage rich tissue	IRAK1, TRAF6 and NF-κB related inflammatory signaling	Reduced inflammatory signaling and accelerated diabetic wound closure	Candidate marker to test under repeated TLR activation from mixed microbiota and biofilm	Taganov et al., 2006; Li et al., 2023b
miR-223	Mesenchymal stem cell EVs in cutaneous wound models	Macrophages and myeloid cells	pknox1 and NLRP3 related inflammatory control	Promoted M2 macrophage polarization and wound repair	Relevant to prolonged macrophage activation and neutrophil-rich inflammation	Johnnidis et al., 2008; Haneklaus et al., 2013; He et al., 2019
miR-21	M2 macrophage-derived EVs	Endothelial cells	PTEN, AKT and mTOR signaling	Enhanced angiogenesis and full-thickness wound repair	Links reparative macrophage state with vascularized granulation tissue	Lyu et al., 2022
miR-21-3p	Human umbilical cord blood derived EVs	Endothelial cells and fibroblasts	Angiogenic and fibroblast activation programs	Promoted angiogenesis, fibroblast function and wound closure	Supports simultaneous vascular and stromal repair in fragile wound beds	Hu et al., 2018
miR-126	BMMSC EVs overexpressing miR-126	Endothelial cells	VEGF and angiopoietin related responses, SPRED1 and PIK3R2 biology	Improved endothelial proliferation, migration and angiogenesis in diabetic wounds	Candidate to test for restoring perfused granulation under hypoxia and inflammation	Fish et al., 2008; Tao et al., 2017
miR-132	Engineered adipose derived stem cell EVs	Macrophages and endothelial cells	NF-κB related immune signaling and angiogenic pathways	Reduced inflammation, promoted M2 polarization and enhanced angiogenesis	May coordinate immune relaxation and vascular repair after perianal infection control	Ge et al., 2023
miR-145-5p	Placenta mesenchymal stem cell extracellular vesicles	Fibroblasts	CDKN1A with Erk and Akt related signaling	Reduced fibroblast senescence and improved diabetic wound healing	Relevant to fibroblast exhaustion and delayed granulation	Su et al., 2023
miR-4645-5p	Hypoxic BMSC derived EVs	Keratinocytes	Autophagy related epithelial repair	Supported keratinocyte repair and wound closure in diabetic wounds	Candidate cargo to test for epithelial-edge repair in moist perianal wounds	Shi et al., 2024
miR-24-3p	Diabetes associated pathological EVs	Endothelial cells	PIK3R3 related angiogenic signaling	Suppressed angiogenesis and delayed repair, while inhibition restored healing	Candidate adverse cargo to test when infection and stress impair vascular repair	Xu et al., 2020
miR-155	Inflammatory miRNA cargo and inhibitor loaded EV strategies	Immune cells and repair cells	Inflammatory signaling and FGF related epithelial support	Excessive activity may delay diabetic wound healing, while inhibition can improve repair	Candidate inflammatory cargo to monitor in persistently inflamed perianal wounds	O'Connell et al., 2007; Gondaliya et al., 2022
miR-93-3p	Wound-exudate vesicle-enriched fraction after Wugu Qilin Ointment treatment	THP-1-derived macrophages (subsequent direct miRNA transfection)	EIF4EBP1	Treatment-associated wound-fluid miRNA change; mimic/inhibitor and luciferase experiments supported macrophage-polarization effects	Direct human perianal association, but patient-derived EV transfer and EV/cargo-dependence were not demonstrated	Zhang et al., 2025

**TABLE 2:** Critical appraisal of completed primary studies directly relevant to perianal or infection-relevant natural-product and extracellular-vesicle evidence.

Study/intervention	Material identity/composition and characterization	Model/comparator	Exposure/duration	What the data support	Methodological credibility/key limitation
Qu et al., 2022 Cuyuxunxi prescription Level 1	Cuyuxunxi (CYXX), 93 g/prescription: *Reynoutria japonica* Houtt. [Polygonaceae], 30 g; *Taraxacum mongolicum* Hand.-Mazz. [Asteraceae], 30 g; *Rhus chinensis* Mill. [Anacardiaceae], 15 g; *Sophora flavescens* Aiton [Fabaceae], 9 g; *Angelica sinensis* (Oliv.) Diels [Apiaceae], 9 g. The 93-g formulation is reported in the CYXX formulation paper and rat study (Qu et al., 2019; Zhang et al., 2022). The 2022 human report did not document voucher specimens or a chemical fingerprint	10 patients after anal-fistula surgery; randomized CYXX rinse vs. normal saline; tissue sampled on postoperative day 7	Topical postoperative wound rinsing. The human report does not clearly state the administered rinse concentration or volume	Short-term human perianal tissue response: less inflammatory-cell infiltration and 472 repair/inflammation-related DEGs	n = 10; day-7 surrogate tissue endpoints only; no robust healing-time, recurrence, or safety endpoint; study-specific botanical authentication and administered exposure incompletely reported; no EV/miRNA causal test
Nazari et al., 2022 hUC-MSC-derived EVs Level 1	hUC-MSC-derived EV preparation; sequential centrifugation/ultracentrifugation; TEM, dynamic light scattering, and CD9/CD63/CD81 by flow cytometry/Western blot. Characterization predates the fuller MISEV2023 control framework	Open-label phase I; 5 patients with refractory Crohn’s perianal fistulas; clinical examination and MRI; no randomized comparator	Single local injection of 5 mL EV solution at 50 μg/mL into tissue surrounding the fistula tract; 6-month follow-up	4/5 improved and 3/5 achieved complete healing; no reported local or systemic adverse events	Exceptionally small uncontrolled study; primarily safety/feasibility evidence. No natural product or specific miRNA analysis; Crohn’s etiology is anatomically direct but not equivalent to cryptoglandular/postoperative infection
Pak et al., 2023 Placenta-MSC-derived EVs Level 1	Placenta-MSC-derived EV preparation; ultracentrifugation reported; clinical and MRI follow-up. The report does not provide sufficient detail to determine the exact particle/mass dose or treatment-session parameters	Single-arm phase I; 11 non-Crohn’s patients with complex perianal fistulas; no concurrent control arm	Local EV administration in the operating room; 6-month follow-up	10/11 showed clinical improvement and 5/11 complete tract resolution; no acute or delayed injection-related complications reported	Small single-arm study; dosing detail incompletely reported; no natural product or miRNA causality. Provides a feasibility/safety signal, not comparative efficacy
Hadizadeh et al., 2024 hUC-MSC-derived EVs Level 1	hUC-MSC-derived EV preparation; clinical examination, MRI, serial tissue sampling and immunohistopathology	Nonrandomized, nonblinded single-center phase II Crohn’s perianal-fistula study; 23 enrolled, 20 completed; no randomized/blinded comparator	5 mL EV solution injected into fistula tracts three times at 2-month intervals; followed monthly for 6 months after the last injection	12/20 patients achieved complete closure; 30/43 tracts closed; local inflammation decreased and regenerative histology improved	Early-phase uncontrolled evidence; related clinical development network to the phase I Crohn’s study; no natural product or specific miRNA mechanism. Crohn’s etiology limits transfer to infection-driven wounds
Wang et al., 2026b Compound Phellodendron Decoction Level 1	Multi-herbal CPD; randomized trial report describes standardized topical preparation, but full batch-level chemical standardization remains limited	Randomized, double-blind, placebo-controlled post-fistulotomy trial; n = 60 (30/30) plus primary human neutrophil assays	Daily topical ultrasonically nebulized CPD or placebo; postoperative follow-up to complete healing	Supports a clinically meaningful reduction in healing time with concordant reductions in NET-related tissue/primary-neutrophil readouts	One of the strongest direct clinical designs. Mechanistic experiments support NET-associated activity but do not prove ROS/PAD4/NETosis is the necessary mediator of clinical benefit
Zhang et al., 2025 Wugu Qilin Ointment Level 1	Mixed-origin ointment containing *Chrysomya megacephala*, Daemonorops draco, Coptis chinensis and Arnebia euchroma; 2:2:2:1 ratio reported	15 low-anal-fistula postoperative patients randomized to Wugu Qilin, Kangfuxin or Vaseline (n = 5/group); wound-exudate vesicle/miRNA profiling plus THP-1 assays	Twice-daily dressings until complete healing; wound exudate used for vesicle-enriched miRNA analysis	Direct human treatment-associated wound-fluid vesicle-enriched miRNA signal; miR-93-3p/EIF4EBP1 showed functional effects in macrophage assays	Very small cohort. EV identity was not established with a full MISEV2023 panel, and recipient-cell experiments used direct miRNA transfection rather than patient-EV transfer/blockade; no EV-mediated causal chain
Yang et al., 2026a Modified Hu-Lian-Bi-Guan decoction Level 1	Multi-herbal oral/topical traditional formulation as reported in the trial; material standardization less complete than pharmaceutical preparations	Randomized controlled trial in complex anal fistula; n = 56 (28/28), with allocation concealment; serial wound assessments and tissue analysis	Postoperative add-on treatment with follow-up through wound healing	Supports faster wound healing and improved serial wound-healing rates with TGF-beta/Smad-related tissue changes	Small single-center study and incomplete blinding; pathway changes are treatment-associated and do not establish mechanistic necessity
Zhao et al., 2026 Radix Arnebiae oil Level 1	Topical Radix Arnebiae oil; comparator ointments included MEBO and petrolatum. Botanical/chemical batch standardization remains incompletely reported	Randomized three-arm study after perianal-abscess incision/drainage; n = 90 (30/group); sealed-envelope allocation and blinded outcome assessment	Topical treatment from postoperative day 1 until complete healing	Supports shorter healing and improved pain/exudation with lower pyroptosis-related biomarkers	Visible topical preparations limited participant blinding. Caspase-1/IL-1beta/IL-18 changes do not establish pyroptosis as a necessary mechanism
He et al., 2026 Calvatia lilacina spores Level 1	Calvatia lilacina spore preparation with UV, GC, HPLC and UPLC-MS characterization	20 postoperative anal-fistula patients with granulation-tissue single-cell RNA sequencing plus macrophage/fibroblast co-culture and pathway assays	Topical spore treatment; clinical tissue sampled for single-cell and molecular analyses	Supports direct human tissue-level effects and macrophage-fibroblast interaction changes with JAK2/STAT3-associated experimental support	Very small clinical sample; single-cell communication and pathway associations do not by themselves establish treatment efficacy or pathway necessity
Chen et al., 2026 Cayratia japonica hydrogel Level 1	Cayratia japonica hydrogel with metabolite/network analyses; luteolin-7-glucuronide proposed among candidate metabolites	Prospective randomized anal-fistula postoperative study (n = 81) plus multi-omics, network pharmacology and BMP4-focused experiments	Local hydrogel applied postoperatively; wound closure compared with standard care	Supports a direct clinical wound-healing signal and provides candidate phase-specific molecular hypotheses	Network/docking outputs are hypothesis-generating; public skin-omics and BMP4 experiments do not prove BMP4 mediates the clinical effect
Yang et al., 2026b Compound Galla chinensis lotion Level 1	Compound Galla chinensis fumigation-washing formulation; full modern chemical standardization not reported	Randomized postoperative anal-fistula study; n = 112 (56/56); potassium-permanganate sitz bath comparator; wound-flora 16S analysis	Postoperative fumigation-washing; before/after tissue and microbial assessments	Supports a direct clinical signal with associated inflammatory/immune and microbiome-composition changes	16S relative abundance does not establish absolute bacterial clearance or mediation of healing; blinding and material standardization are limited
Li et al., 2009 Qiyu oil gauze Level 1	Qiyu oil gauze; exact botanical composition and modern chemical/batch standardization were not adequately detailed in the indexed report	60 low-anal-fistulectomy patients assigned by hospitalization sequence to Qiyu or Vaseline gauze	Daily wound packing until healing; granulation tissue assessed on postoperative day 5	Supports shorter healing time with higher VEGF/bFGF and capillary counts; EGF was not significantly different	Non-random sequence allocation and older reporting standards increase selection-bias risk; growth-factor changes are associative
Yang et al., 2017 Daphne genkwa root extract Level 1	Daphne genkwa root extract; clinical material characterization and exposure standardization are incompletely detailed	60 postoperative anal-fistula patients randomized to external extract or control; paired TNF-alpha-damaged human skin fibroblast experiments	External postoperative treatment; granulation tissue and fibroblast molecular assays	Supports a human healing signal and collagen/fibroblast-related biological activity	Cell MEK/ERK activation does not prove that pathway mediates the human clinical effect; extract characterization is limited
Cai et al., 2023a Zibai ointment Level 1	Zibai ointment; detailed modern chemical standardization not reported	Randomized postoperative anal-fistula study; n = 90 (45/45), Zibai ointment versus petroleum jelly	Topical postoperative application with serial healing and tissue apoptosis assessment	Supports improved wound healing with treatment-associated Bcl-2/Bax/TUNEL changes	Biomarker changes are associative; limited blinding and formulation standardization constrain mechanistic inference
Xiong et al., 2025 Neibu Huangqi Youhua formula + Kangfuxin Level 1	Oral/internal traditional formula used together with Kangfuxin wound care	Randomized postoperative anal-fistula study; n = 160; Kangfuxin alone versus formula plus Kangfuxin	Add-on postoperative regimen with serial wound, pain and inflammatory assessment	Supports an incremental benefit of the combined regimen over Kangfuxin alone	Cannot isolate the formula’s absolute effect because the intervention is add-on therapy; blinding and mechanistic causality are limited
Deng et al., 2022 Compound Phellodendron Decoction Level 1	Five-material decoction: Phellodendri Chinensis Cortex 30 g, Sophorae flavescentis Radix 25 g, Salviae miltiorrhizae 15 g, Bletillae rhizoma 10 g, Angelicae dahuricae Radix 20 g; 100 g crude drug yielded 400 mL (0.25 g/mL). Microbial quality check reported; no voucher-authentication or chemical fingerprint reported	100 post-fistulotomy patients; random-number-table allocation, 50/50; CPD fumigation/sitz bath vs. warm water; no blinding reported	200 mL decoction added to 1.8 L warm water; 12 min once daily from postoperative day 2	Supports a direct human wound-healing signal with lower pain/edema/exudation, shorter healing time, cytokine changes and more granulation-tissue capillaries	Randomization reported but blinding absent; biomarker changes are associative. Related research programme to the 2026 CPD trial, so not independent replication; limited botanical/chemical standardization
Pan et al., 2025 Zhenshi Burn Ointment Level 1	Named traditional topical ointment. The accessible primary report describes the formulation as Zhenshi Burn Ointment but does not provide a complete botanical/mineral composition or chemical fingerprint in the English abstract	60 patients with low simple anal fistula and damp-heat downward syndrome; randomized 30/30; ointment vs. sterile Vaseline gauze	Topical treatment for 30 days; serial wound assessment through day 30	Supports faster closure, improved granulation/histology and increased collagen/growth-factor and p-MEK/p-ERK readouts	Syndrome-selected single-center cohort; blinding not reported; incomplete accessible material characterization. MEK/ERK activation is treatment-associated, not a necessity/rescue demonstration
Chen and Guo, 2025 Herbal sitz-bath formula Level 1	Ten-material formula: Astragali Radix 30 g, Phellodendri Cortex 12 g, Platycodonis Radix 15 g, Glycyrrhizae Radix 3 g, Coicis Semen 30 g, Angelicae Sinensis Radix 12 g, Paeoniae Radix Rubra 9 g, Chuanxiong Rhizoma 9 g, Corydalis Rhizoma 15 g, Polygonum cuspidatum 15 g; centrally prepared	80 patients after low perianal-abscess surgery; randomized 40/40; adjunctive medicated fumigation/irrigation vs. matched warm-water procedure; wound-exudate 16S sequencing	20 min twice daily for 7 days; day-1/day-8 sampling; final sequencing cohorts 38 treatment and 36 control	Supports treatment-associated changes in wound microbial community structure and relative abundance, including lower *Enterococcus* and higher diversity	16S relative abundance does not establish absolute pathogen burden, viability, metagenomic function, or microbial mediation of healing. Clinical and sequencing endpoints are partly surrogate
Yu et al., 2025 Bismuth subgallate-borneol wound-care ointment Level 1	Proprietary ointment containing 4.5% bismuth subgallate and 0.7% d-borneol in a Vaseline base	46 patients with perianal abscess or anal fistula; prospective randomized 23/23; ointment plus conventional care vs. conventional care alone	Topical adjunct during postoperative follow-up; wound area quantified by ImageJ at days 4, 7 and 14	Supports faster granulation and a higher wound-healing rate by day 14	Mixed diagnostic population and small sample. Control care included traditional Chinese medicine injection; no significant healing-rate difference on days 4 or 7, so benefit was time-dependent and incremental
Lu et al., 2025 Jiedu Shengji ointment Level 1	Named Jiedu Shengji ointment used with Vaseline gauze; the indexed report does not provide complete study-specific formulation authentication or chemical characterization	Retrospective cohort of 60 postoperative anal-fistula patients; 30 ointment + Vaseline vs. 30 Vaseline according to treatment received	Dressing treatment with assessment at 7 and 14 days and clinical healing outcomes	Supports an association with shorter healing, lower wound scores and changes in IL-6/SAA, TGF-beta and EGF.	Retrospective nonrandom treatment allocation creates selection and residual confounding; biomarker changes cannot establish mechanism
Du et al., 2024 Phased Chinese-herbal sitz bath Level 1	Two-stage traditional decoction strategy: both groups initially received the same Qingre Zaoshi Zhitong formula; the staged group later switched to a Yiqi Fuzheng formula. Full modern chemical standardization was not reported in the indexed record	200 postoperative complex anal-fistula patients randomized 100/100; continuous early formula vs. stage-adapted formula sequence	Sitz bath twice daily for 6 weeks	Supports a shorter mean incision-healing time (22.96 vs. 26.19 days) and improved symptom/growth-factor outcomes with stage-adapted treatment	Does not estimate herbal treatment versus standard care because both groups received herbs; it tests timing/formula sequencing. Blinding and material standardization are limited
Wu et al., 2024 Yanghe decoction Huacai Level 1	Named oral traditional formula evaluated in a syndrome-defined slow-healing postoperative population; full study-specific chemical fingerprinting was not reported	120 patients with slow-healing wounds after anal-fistula surgery; randomized intervention/control comparison	Postoperative oral intervention with serial clinical and growth-factor assessment	Supports improved wound-repair outcomes accompanied by bFGF, TGF-beta1 and EGF changes	Syndrome-selected population limits generalizability; growth-factor changes are associative and the accessible report does not establish pathway necessity
Tsai et al., 2026 Micronized purified flavonoid fraction (MPFF) Level 1 (contextual)	Standardized oral micronized purified flavonoid fraction; included as a natural-product-derived pharmacological comparator rather than an ethnopharmacological preparation	106 randomized after fistulotomy/fistulectomy; 104 analyzed, 52/52; MPFF vs. control	1 g twice daily; early postoperative symptom tracking and 3-month wound/complication assessment	Supports lower postoperative pain and fewer dressing changes	No significant difference in wound healing, analgesic use, complications or recurrence. Demonstrates that symptomatic benefit does not necessarily translate into faster healing
Zhang et al., 2022 Cuyuxunxi prescription Level 2	Same five-botanical CYXX formula listed in full in the Qu et al. row above (93 g total; individual amounts 30, 30, 15, 9, and 9 g). Formula amounts were reported, but study-specific voucher authentication and chemical fingerprinting were not	54 SD rats with simulated postoperative anal-fistula wounds; saline vs. rivanol vs. CYXX.	Decocted twice in 500 mL water; two 125-mL extracts combined to 250 mL (3.72 g crude drug/mL); external dressing once daily for 14 days	Improved early wound-healing rate and several repair-related growth-factor readouts	Single animal model with surrogate molecular outcomes; wound-tissue VEGF was not significantly different among groups at reported time points; no EV/miRNA analysis; formulation chemistry limits reproducibility
Li et al., 2026 Xiangpi Shengji Ointment (XPSJG) Level 2	In the study’s ETCM-based analysis, XPSJG was represented by a mixed-origin six-material set: *Angelica sinensis* (Oliv.) Diels [Apiaceae] (Dang Gui); Testudinis Carapax et Plastrum (Gui Jia); beeswax/Feng La with *Apis cerana* Fabricius and *Apis mellifera* Linnaeus; Gypsum fibrosum (Shi Gao); Crinis carbonisatus (Xue Yu Tan); and Smithsonite (Lu Gan Shi). UPLC-QTOF-MS profiling of the aqueous extract and HPLC comparison with the complete formulation were reported; the article does not report exact material amounts or ratios, manufacturer, batch, or study-specific authentication/voucher information	Integrated network/transcriptomic analysis, macrophage/fibroblast experiments and an anal-fistula animal model; NF-κB inhibitor challenge	Aqueous extract 0, 125, 250 and 500 mg/L *in vitro*; topical *in-vivo* treatment emphasized the early healing phase	Supports stage-dependent macrophage-fibroblast crosstalk and NF-κB/HIF-1α/VEGF-associated healing in an anatomically relevant model	Network/transcriptomic discovery remains hypothesis-generating. Species/source authentication and provenance are not equally documented for all zoological/mineral materials; no EV/miRNA endpoint
Jian et al., 2026 XPSJG + Qingre Zaoshi compound (Coptis–Phellodendron extract) Level 2	The article describes XPSJG combined with Qingre Zaoshi compound/CGE, an extract of *Coptis chinensis* Franch. [Ranunculaceae] and *Phellodendron amurense* Rupr. [Rutaceae]. Taxonomic nomenclature for both plant species follows Kew databases. The article does not report the Coptis:Phellodendron extract ratio, complete mixed-formulation provenance, or study-specific voucher/authentication details	Anal-fistula rat model plus LPS-THP-1 macrophage and HUVEC experiments; proteomics/metabolomics; NF-κB overexpression	Topical XPSJG plus Qingre Zaoshi compound/CGE *in vivo*; the exact formulation ratio and delivered exposure were not reported in the article	Supports reduced inflammation, M2-associated macrophage shift, VEGF/angiogenesis and NF-κB pathway involvement	Related research group/model platform to Li et al., 2026; not fully independent replication without confirmation. Mixed formulation; no EV/miRNA endpoint
Lu et al., 2024 hUC-MSC-derived EVs Level 2	hUCMSC-EVs; differential ultracentrifugation plus purification column; TEM, nanoparticle tracking, total protein and EV-marker Western blot; ARRIVE 2.0 stated	40 male SD rats; complex perianal-fistula model with blank, model and low/medium/high EV-dose groups; ultrasound, histology and molecular outcomes	Dose-ranging local EV treatment; 10 μg/100 μL produced the strongest reported effect	Direct perianal evidence for improved healing, collagen, angiogenesis and inflammatory regulation with HIF-1α/TGF-β/Smad-associated changes	Animal study; no natural product or defined miRNA causality. Pathway activation is associative rather than a necessity/rescue demonstration; model does not reproduce the full polymicrobial/repeated-fecal-exposure ecology
Li et al., 2025 MSC-EV nanofiber-hydrogel composite (EV-NHC) Level 2	MSC-EVs characterized by TEM/NTA; nanofiber-hydrogel rheology and EV retention/release assessed	Rat Crohn’s-disease perianal-fistula model; surgery alone, NHC, MSC-NHC, tissue-graft comparator and EV-NHC; MRI and tissue endpoints	3 × 10^11 EVs incorporated into each NHC; outcomes assessed about 20 days after treatment	EV-NHC produced the strongest MRI-defined healing, prolonged local EV retention, reduced inflammation and supported macrophage polarization/neovascularization	Crohn’s inflammatory fistula model rather than cryptoglandular infection; no natural product or specific miRNA mechanism. Biomaterial and EV effects are partly coupled
Pang et al., 2025 Danggui Buxue Tang Level 2	Decoction prepared from Astragalus membranaceus and Angelica sinensis	Fistula-like mouse wound model; control vs. DBT; day 1/day 7 single-cell RNA sequencing	DBT administered after model establishment; serial wound assessment	Supports faster healing with a treatment-associated shift from M1-like to M2-like macrophage states and OPN/PI3K/Akt/eNOS-related signals	Single-cell trajectories are high-resolution but observational; pathway necessity was not tested by inhibitor/rescue
Wang et al., 2026a Danshen/Huanglian-loaded gelatin/Bletilla striata gum dressing Level 2	Composite herbal/biomaterial dressing with Danshen and Huanglian loaded into gelatin/Bletilla striata gum	Rat anal-fistula model with GB, Danshen-GB and Danshen/Huanglian-GB comparators; primary fibroblast experiments with si-TGF-beta	Two-week local dressing treatment	Supports direct anal-fistula wound healing, collagen/blood-flow/angiogenic benefits and TGF-beta/Smad involvement with cellular knockdown evidence	Multicomponent carrier and herbal effects are coupled; si-TGF-beta validation was *in vitro* rather than an *in-vivo* rescue
Xiao et al., 2026 pH-responsive berberine/EGCG hydrogel Level 2	Acid-responsive OCMC/CMCS hydrogel with berberine and epigallocatechin gallate; drug purity and release properties characterized	Rat anal-fistula model with biomaterial/drug comparisons; antibacterial, oxidative-stress, macrophage, angiogenesis and matrix outcomes	Sequential release designed for early BBR and sustained EGCG activity	Supports stage-oriented delivery and direct anal-fistula efficacy	Carrier, BBR and EGCG effects are partly coupled; pathway readouts do not establish a single necessary molecular target and no EV/miRNA mechanism was tested
Fang et al., 2026b Zuoqing granule Level 3	Zuoqing granule; corrected article consulted	Dorsal full-thickness rat wound with daily fecal-supernatant contamination; n = 10/group; low/medium/high-dose ZQG plus neutrophil Nox4 knockdown experiments	Daily topical gauze treatment for 7 days	Supports dose-responsive suppression of bacterial burden, inflammation and NETosis with Nox4-related cellular evidence	Despite the article’s model label, the wound was dorsal and not anatomically perianal. No *in-vivo* direct NET-disruption rescue; classify as Level 3
Fang et al., 2026a Portulaca oleracea extract Level 3	Portulaca oleracea L. extract; topical exposure 100 μg/mL	Rat dorsal full-thickness infected wound with fecal contamination; n = 6/group	Daily extract-coated gauze for 14 days	Supports improved histological/inflammatory outcomes with higher CD34 and VEGFA-related markers	Not a perianal wound; small groups; VEGF involvement is based on associative expression data rather than causal perturbation
Zhang et al., 2024a Huanglian ointment (HLO) Level 3	Primary-study formula: Coptis Rhizome 11.175 g; Chinese Angelica 18.625 g; Amur Corktree Bark 37.250 g; Rehmannia Root 11.175 g; Turmeric 11.175 g (89.4 g total), with 447 g sesame oil. The primary paper reports crude-drug names but not voucher-authenticated botanical species; unreported taxa were therefore not inferred from other HLO formulations	24 SD rats; dorsal S. aureus-infected wound used to imitate poor healing after anal-fistula surgery; control, infected model, potassium permanganate and HLO groups (n = 6/group)	Soaked 24 h and fried at 200 °C for 4 h; final HLO 0.2 g/g; once daily for 14 days	Supports activity of the quantitatively described HLO formulation in an infected open-skin wound and generates proteomic/metabolomic hypotheses	Not anatomically perianal; small n. Species-level identity/authorities/families, voucher authentication and study-specific chemical fingerprint are absent in the primary report. This constitutes an ethnopharmacological limitation; omics associations do not prove causal targets; no EV/miRNA analysis
Yin et al., 2022 Dracorhodin perchlorate (DP)-loaded adhesive hydrogel Level 3	Defined DP-loaded biomaterial; hydrogel chemistry characterized by FTIR/1H-NMR and physical testing; antibacterial, cytocompatibility and hemocompatibility assays	1.5-cm full-thickness rat dorsal wounds; *E. coli* infection followed by repeated daily contamination to simulate fecal exposure; hydrogel comparison groups	Initial *E. coli* 10^8 CFU/mL; 200 μL hydrogel after infection; tissue assessed on days 7 and 14	Supports wet-adhesive, antibacterial and regenerative performance under repeated bacterial contamination	Despite perianal framing, wound was dorsal. All rats received postoperative antibacterial drugs, confounding antibacterial attribution; carrier materials are biologically active; no EV/miRNA endpoint
Li et al., 2023c Berberine-HCl liposome gel Level 3	Berberine-HCl liposomes/thermosensitive gel; concentration-response biofilm testing; liposome and gel characterization	*S. aureus* biofilm assays and a mouse infected-wound model; free berberine, liposome/gel and vehicle comparators	12.5–200 μg/mL for 24-h biofilm testing; gel 200 μg/mL; mouse wounds treated for 14 days	Supports formulation-level antibiofilm activity and improved healing in an infected skin wound	Mouse skin is not perianal; commercial berberine-HCl identity/purity less fully characterized than the formulation; PAINS/promiscuity cautions remain for target-level claims; no EV/miRNA causality
Cai et al., 2023b Eucalyptus essential-oil nanoemulsion hydrogel Level 3	Eucalyptus essential oil (EEO) nanoemulsion/hydrogel. The primary report does not establish the Eucalyptus species, voucher/authentication record or a sufficiently detailed chemical fingerprint; species-level identity therefore remains unresolved	*S. aureus* biofilm assays and an infected cutaneous wound model	EEO nanoemulsion MIC 15 mg/mL and MBC 25 mg/mL; biofilm testing at 2×MIC; *in-vivo* hydrogel treatment	Supports antibiofilm and wound-healing activity of a characterized delivery system in infected skin	High local concentrations and nonperianal model limit translation. Species-level botanical identity remains unresolved, which limits ethnopharmacological reproducibility; no EV/miRNA mechanism

Scope. this table contains completed original studies carrying the principal clinical, anatomically perianal, or infection-relevant claims. Evidence proximity (Levels 1-3) is reported separately from methodological credibility; no composite quality score is assigned. Randomization, blinding, comparator quality, sample size, material characterization, exposure, model fidelity, EV, characterization, causal perturbation and replication are assessed narratively in the final column. Protocols, reviews, meta-analyses and guidelines are used for context but are not mixed with completed primary studies; single case reports are discussed in the text as case-level signals rather than tabulated beside trials.

Evidence level follows Section 2.3. Level 1 = completed human perianal/anorectal study; Level 2 = *in vivo* perianal/anal-fistula model; Level 3 = *in vivo* infected-wound model. Level indicates clinical or anatomical proximity, not methodological quality. For identifiable plant taxa, accepted names, author citations and families were checked against the Royal Botanic Gardens, Kew Medicinal Plant Names Services and Plants of the World Online. Materials lacking species-level identity, authentication, chemical fingerprinting or reproducible exposure in the primary report are retained with that uncertainty stated as a limitation.

**TABLE 3:** Critical appraisal of mechanistic bridging primary studies for representative metabolites, extracellular vesicles, and miRNA-related wound repair.

Study/intervention	Model and comparator	Exposure/duration	Characterization and controls	What the data support	Methodological credibility/key limitation
Chen et al., 2016 Baicalein Level 5	Clinical *S. aureus* isolates and biofilm assays; vancomycin/clarithromycin comparators	16, 32, 64 μg/mL (approximately 59, 118, and 237 μM for baicalein); 3- and 7-day biofilms; 12-h combination treatment; 16 μg/mL inactive for major biofilm endpoints	Baicalein purity >98% by HPLC; MIC/growth curves; CFU, crystal violet, fluorescence, SEM/CLSM, virulence and RT-qPCR assays; repeated experiments	Concentration-dependent antibiofilm/anti-virulence phenotype and antibiotic-adjuvant potential *in vitro*	No host tissue or wound model; pharmacologic exposure at a contaminated wound is unknown. Assay-promiscuity caution applies; does not establish clinical anti-infective efficacy or EV/miRNA activity
Ouyang et al., 2016 Quercetin Level 5	*Pseudomonas aeruginosa* PAO1 quorum-sensing/biofilm model	16 μg/mL (approximately 53 μM quercetin) for key transcriptional analyses; bacterial growth not measurably inhibited	MIC/growth-curve controls; biofilm and virulence-factor assays; lasI/lasR/rhlI/rhlR transcription assessed	Supports an *in vitro* anti-virulence/QS phenotype independent of gross growth inhibition	Single bacterial *in vitro* system; no tissue, wound or exposure validation; quercetin is assay-promiscuous, so target-specific interpretation remains low confidence
Kim et al., 2023 Baicalin Level 4	Human epidermal keratinocyte scratch model and mouse ischemia-reperfusion pressure ulcers	100 μg/mL (approximately 224 μM baicalin) in keratinocytes (6–48 h); 100 mg/mL topical baicalin in mice with serial assessment to day 10	CCK-8 cytotoxicity assay; wound area, H&E/Masson, inflammatory cytokines and multiple growth factors	Supports pro-healing activity in a noninfected chronic-wound model with both cellular and *in vivo* observations	Very high topical concentration; pressure-ulcer biology differs from contaminated perianal wounds. No EV/miRNA causality; broad pathway claims should be interpreted cautiously
Gopalakrishnan et al., 2016 Quercetin Level 4	Adult Wistar rats; 2 × 2-cm dorsal full-thickness excision; control vs. quercetin	0.1% quercetin ointment twice daily for 14 days; tissue assessed on days 3, 7, 11, and 14	Wound area, histology, microvessel density, TNF-α, IL-10, VEGF, TGF-β1 and CD31	Supports wound-level pro-repair and angiogenesis-associated effects beyond a single *in vitro* assay	Noninfected dorsal wound; no perianal anatomy, biofilm, EV or miRNA analysis. PAINS/promiscuity means pathway association should not be equated with a single molecular target.
Ding et al., 2022 Resveratrol Level 4	STZ-diabetic C57/B6 mouse wounds plus THP-1 macrophage model	10 μmol/L resveratrol around the wound; 15-day mouse experiment; 10 μmol/L in THP-1 cells	*In vivo* closure and skin structure plus macrophage-polarization and cytokine readouts	Supports an *in vivo* association between resveratrol exposure, M2-associated responses and improved diabetic wound healing	Diabetic but noninfected, nonperianal model; local exposure may not reflect clinical formulation pharmacokinetics; no EV/miRNA causal analysis and assay-promiscuity caution remains
Zhu et al., 2022 Resveratrol nanoparticles + platelet EVs Level 4	Composite GelMA/SFMA hydrogel tested in HUVEC/macrophage assays and diabetic mouse wounds	Resveratrol loaded into mesoporous silica nanoparticles and combined with platelet-derived EVs in sustained-release hydrogel; wound treatment in diabetic mice	Biomaterial characterization, cytotoxicity/biocompatibility, inflammatory markers, tube formation, angiogenesis and wound closure	Supports co-delivery of a natural metabolite formulation and EVs as complementary anti-inflammatory/pro-angiogenic therapy	Does not show that resveratrol modifies EV cargo; EVs and resveratrol are co-formulated rather than mechanistically linked. Noninfected diabetic skin model; no miRNA causality
Li et al., 2023a Curcumin-loaded macrophage-derived EVs Level 4	High-glucose HUVEC assays and diabetic rat skin-defect model	Curcumin-loaded macrophage-derived EVs (Exos-cur) were tested in high-glucose endothelial assays and locally in diabetic rat wounds; outcomes were assessed through the experimental healing period. The article does not report a complete delivered curcumin/particle dose, limiting dose-level interpretation	EV-based carrier; endothelial proliferation/migration/angiogenesis, ROS/mitochondrial assays, histology, Western blot and immunofluorescence	Supports EV-assisted curcumin delivery with wound-level anti-inflammatory, antioxidant and pro-angiogenic effects	Curcumin is assay-promiscuous; carrier delivery mitigates but does not remove target-specificity concerns. No evidence that curcumin altered EV-associated miRNA cargo or that miRNA mediated the wound effect
Xiong et al., 2022 AS-IV-conditioned EPC EV-associated miR-21 Level 5	EPC-derived EVs applied to 33-mM high-glucose-damaged HUVECs	AS-IV pretreatment of EPCs; recipient-cell high-glucose injury; miR-21 mimic/manipulation and PTEN rescue framework	Differential centrifugation, electron microscopy, nanoparticle tracking, qPCR/WB, luciferase target assay, miRNA manipulation and PTEN overexpression	Strongest cargo-level evidence in this review that a natural metabolite can condition donor cells and alter an EV-miRNA/target axis	Cell-only model; no wound, infection or perianal outcome. EV characterization should be judged against MISEV2023, and *in vivo* necessity of miR-21 remains unproven
Xiong et al., 2023 AS-IV-conditioned EPC-derived EVs Level 4	EPC assays and STZ type-I diabetic rat wound model; EPC-derived EVs vs. AS-IV-EPC-derived EVs; rapamycin and wortmannin mechanistic comparators	EPCs exposed to 50, 100, or 200 mg/L AS-IV (approximately 64, 127, and 255 μM for astragaloside IV); 100 mg/L produced the strongest EPC/EV response; EVs then tested in diabetic wounds	CD9/CD63/CD81, TEM, EV quantification; wound closure, collagen, BrdU, VEGF, CD31, α-SMA, CD45; pathway inhibitor reversal	Supports donor-cell conditioning by AS-IV, EV involvement, *in vivo* wound efficacy, and PI3K/AKT/mTOR-associated mechanism	Does not identify a necessary miRNA cargo in the wound experiment; noninfected diabetic wound; morphology/positive markers alone are not a complete MISEV2023 characterization
Huang et al., 2021 Ginsenoside Rg1 Level 4	High-glucose HUVEC model plus diabetic-foot-ulcer animal model	Rg1 treatment in high-glucose HUVEC and diabetic-foot-ulcer models with miR-489-3p gain/loss experiments; the exact Rg1 exposure was not reported in the article, limiting pharmacological dose appraisal	MTT/apoptosis/oxidative-stress assays; dual-luciferase validation of miR-489-3p-Sirt1; miRNA manipulation and *in vivo* wound closure	Supports a natural-metabolite-to-miRNA pathway with stronger target validation than expression association alone	The miRNA mechanism is not EV-mediated; model is diabetic rather than infected/perianal. Accordingly, it supports miRNA plausibility but does not establish EV-mediated regulation
Zhou et al., 2023 Salvianolic acid B hydrogel Level 4	Cell compatibility/migration assays and diabetic wound model using an HA self-healing hydrogel	SAB tested in hydrogels at 0, 1, 2.5, and 5 mg/mL (approximately 0, 1.39, 3.48, and 6.96 mM for salvianolic acid B) with local wound delivery	Hydrogel physical characterization, cell assays, wound closure, inflammation and angiogenesis-related outcomes	Supports dose-formulated local delivery of SAB in a chronic-wound setting	Noninfected, nonperianal model; no EV/miRNA analysis. Isolated-metabolite data cannot be generalized to Salvia botanical preparations without separate characterization
Lee et al., 2016 Ginsenoside Rb1 (not whole Panax extract) Level 5	Cultured human dermal fibroblasts; untreated cells as control	G-Rb1 tested over a concentration range; 10 ng/mL (approximately 9 nM) used for principal fibroblast experiments; 72-h exposure	Cell proliferation, collagen and growth-factor/MMP-TIMP assays	Supports an *in vitro* Rb1 effect on fibroblast proliferation/collagen-related readouts	Article title refers to *Panax ginseng* C.A.Mey. [Araliaceae] extract, but the experimental treatment was isolated G-Rb1. No botanical-extract characterization, wound model, infection, EV or miRNA endpoint; should not be cited as evidence for whole-ginseng efficacy
Xiong et al., 2024b AS-IV-conditioned EPC EV miR-126-3p Level 4/5	AS-IV-treated EPCs, high-glucose HUVECs, and diabetic rat skin wounds; miR-126-3p inhibitor vs. mock	AS-IV donor-cell conditioning followed by EPC-EV treatment; *in* *vitro* and *in vivo* diabetic-wound experiments	EV release/characterization, miR-126-3p inhibition, PIK3R2/SPRED1-related readouts and rat wound outcomes	Supports a natural-metabolite - > donor-cell EV - > defined miRNA/target-axis bridge extending into an *in-vivo* wound model	Diabetic noninfected skin rather than perianal wound; related AS-IV research programme rather than independent replication; *in-vivo* cargo necessity remains incomplete
Dai et al., 2026 Salvia-Astragalus herb-pair DH-EVs hydrogel Level 3/4	Plant/herbal-derived EV-like particles incorporated into hydrogel; bacteria-infected diabetic wound model	Local DH-EV hydrogel treatment; 10% DH-EV formulation showed strongest reported activity	NTA/TEM, lipidomics, small-RNA sequencing, hydrogel characterization, NET/inflammation assays and infected-wound outcomes	Supports a chemically/vesicularly characterized herbal EV-like delivery platform with infected-wound activity and candidate small-RNA cargo	Candidate miRNAs were not individually perturbed *in vivo*; plant/herbal vesicle-like particles are not equivalent to mammalian EVs; diabetic wound is not perianal
Suresh et al., 2025 Mango-ginger-derived nanovesicles Level 4	Keratinocyte scratch assays and STZ-diabetic mouse wounds; hydrogel-formulated plant-derived nanovesicles	Topical nanovesicle hydrogel; porcine-skin penetration also assessed	Plant-derived nanovesicle characterization, migration assays, *in-vivo* healing and FSTL1-associated readouts	Supports plant-derived nanovesicle pro-migratory activity and diabetic-wound repair	Noninfected nonperianal model; FSTL1 association does not by itself establish the active vesicle cargo or causal mediator
Miya et al., 2024 OXY-ExoAloe Level 4	Combined aloe-vera, ginger and neem exosome-like vesicle preparation in diabetic wounds	Topical multicomponent vesicle-like therapy in diabetic wound injury	Standardized mixture with wound, inflammatory, oxygenation and matrix-remodeling readouts	Supports wound-level activity of a multicomponent plant vesicle-like preparation	Multicomponent origin prevents attribution to one plant or cargo; no defined miRNA causality; noninfected nonperianal model
Hu et al., 2018 hUCB-derived EV miR-21-3p Level 4	Endothelial/fibroblast assays plus a cutaneous wound model; UCB-EVs compared with controls and miR-21-3p/target perturbation	Local UCB-EV treatment in skin wounds with parallel *in-vitro* endothelial and fibroblast experiments	EV characterization; specific RNA inhibitors/siRNAs; PTEN and SPRY1 target interrogation; wound, angiogenesis and fibroblast readouts	Supports EV-mediated miR-21-3p as a functional contributor to angiogenesis, fibroblast activity and cutaneous wound repair	Credibility strengthened by cargo and target perturbation across cell and wound models; noninfected cutaneous model and no natural-product exposure limit relevance to perianal infection
He et al., 2019 MSC-EV miR-223 Level 4	MSC transplantation/exosome experiments in cutaneous wounds with macrophage-polarization analyses	MSC/MSC-exosome treatment during cutaneous repair; miR-223-related macrophage experiments	Macrophage-depletion context, EV experiments, miR-223 manipulation and pknox1-related target analysis	Supports an MSC-EV miR-223 pathway promoting reparative macrophage polarization and wound healing	Mechanistic credibility is stronger than expression-only evidence, but the model is noninfected skin and does not test a natural product or perianal anatomy
Lyu et al., 2022 M2-macrophage exosomal miR-21 Level 4	HUVEC angiogenesis assays and full-thickness mouse cutaneous wounds treated with M2-macrophage exosomes	M2-EXO exposure *in vitro* and local treatment in full-thickness wounds	Exosome characterization; miR-21 transfer; PTEN/AKT/mTOR-related assays; angiogenesis and tissue-regeneration endpoints	Supports donor-macrophage EV transfer of miR-21 to endothelial cells with pro-angiogenic wound effects	Direct donor-to-recipient pathway is a strength; absence of infection, perianal anatomy and natural-product conditioning limits translation
Tao et al., 2017 miR-126-overexpressing MSC exosomes + chitosan dressing Level 4	Engineered synovial-MSC exosomes incorporated into chitosan dressing; endothelial assays and diabetic rat full-thickness wounds	Sustained local release of miR-126-enriched exosomes from chitosan dressing during diabetic wound repair	Engineered cargo enrichment, carrier-release characterization, endothelial proliferation/migration/tube formation, angiogenesis and wound closure	Supports sufficiency of miR-126-enriched EV delivery for angiogenesis and diabetic wound repair	Engineered overexpression demonstrates therapeutic sufficiency rather than endogenous disease mechanism; noninfected diabetic skin and no natural-product conditioning
Li et al., 2023b Engineered miR-146a EV silk-fibroin patch Level 4	Engineered placental-MSC exosomes attached to silk-fibroin patch and tested in diabetic wounds	Sustained release of miR-146a-loaded engineered exosomes from the patch through the wound-healing period	Engineered loading/binding system, EV characterization, inflammatory and vascular readouts, IRAK1-focused mechanistic testing	Supports sustained local delivery of miR-146a EVs with reduced inflammation, improved collagen deposition and neovascularization	Strong engineering and target-focused design; diabetic noninfected model, artificial cargo enrichment and no natural-product component limit direct perianal inference
Ge et al., 2023 miR-132-overexpressing adipose-stem-cell exosomes Level 4	Engineered adipose-stem-cell exosomes tested in HUVECs, STZ-diabetic full-thickness wounds and skin-flap models	Local administration of miR-132-enriched exosomes in diabetic wound/skin-flap models	Cargo overexpression, endothelial proliferation/migration, inflammation, macrophage polarization, angiogenesis and NF-kB-related readouts	Supports therapeutic sufficiency of miR-132-enriched EVs for inflammatory control and vascular repair	Engineered cargo and diabetic skin setting limit inference about endogenous perianal EV biology; pathway association is stronger than direct rescue-based necessity testing
Su et al., 2023 P-MSC-EV miR-145-5p/CDKN1A Level 4	High-glucose senescent human dermal fibroblasts plus full-thickness diabetic mouse wounds	P-MSC-EVs at low/high particle concentrations *in* *vitro* and local EV treatment in diabetic wounds	Dose response, miRNA profiling, miR-145-5p inhibitors/mimics, CDKN1A siRNA, Erk/Akt analysis and *in-vivo* miR-145-5p agomir	Supports EV transfer of miR-145-5p as a functional contributor to fibroblast recovery and diabetic wound repair	One of the stronger cargo-to-recipient studies because inhibitor/mimic/target perturbation accompany the wound phenotype; still noninfected diabetic skin and not natural-product linked
Xu et al., 2020 Pathological exosomal miR-24-3p/PIK3R3 Level 4	Diabetes-associated circulating exosomes tested in endothelial cells and wound-repair models	Diabetic exosome exposure with miR-24-3p inhibition and wound-repair assessment	miRNA enrichment, loss-of-function, luciferase target validation for PIK3R3 and endothelial functional assays	Supports a harmful EV cargo in diabetes and shows that miR-24-3p inhibition can restore angiogenic function and improve repair	Loss-of-function and target validation strengthen causality; disease context is diabetic vascular dysfunction rather than infected perianal healing
Gondaliya et al., 2022 MSC exosomes loaded with miR-155 inhibitor Level 4	*In vitro* co-culture and diabetic mouse wound model using inhibitor-loaded MSC exosomes	Local/experimental delivery of miR-155-inhibitor-loaded MSC exosomes during diabetic wound repair	Engineered inhibitor loading, keratinocyte migration, FGF-7 restoration, inflammatory readouts and *in-vivo* wound closure	Supports therapeutic suppression of miR-155 using EV delivery in diabetic wounds	Useful loss-of-function therapeutic model, but inhibitor-loaded exosomes do not establish endogenous EV-miR-155 transfer in perianal wounds and the model is noninfected
Shi et al., 2024 Hypoxic BMSC-EV miR-4645-5p/MAPKAPK2 Level 4	Keratinocyte experiments plus diabetic mouse wound model using hypoxic BMSC-derived exosomes	Hypoxic BMSC-EV treatment with miR-4645-5p and target-pathway manipulation during diabetic wound repair	EV transfer experiments, dual-luciferase target validation, MAPKAPK2-AKT-mTORC1/autophagy assays and wound endpoints	Supports EV-mediated miR-4645-5p regulation of keratinocyte autophagy, proliferation/migration and diabetic wound healing	Target validation and recipient-cell biology strengthen the mechanism; perianal infection and natural-product conditioning were not studied

Scope. Completed original studies carrying the main mechanistic bridging claims are appraised here. Where a study spans cell and *in-vivo* components, both evidence levels are shown. Methodological credibility is judged separately from proximity and is expressed narratively rather than as a composite score. For plant-derived metabolites, dose-response, viability/cytotoxicity controls, orthogonal assays and target perturbation increase confidence; for EV-miRNA, studies, vesicle characterization, cargo manipulation, target validation, loss-of-function and rescue are given greater weight than expression association alone. Plant/herbal vesicle-like particles are appraised separately from mammalian EVs.

Potential assay interference was considered explicitly for frequently assay-promiscuous plant-derived metabolites, particularly berberine, baicalin/baicalein, curcumin, quercetin, and resveratrol. PAINS alerts were treated as a reason for increased evidential caution rather than automatic invalidation. Conclusions based predominantly on a single biochemical or cell-based assay were down-weighted, whereas concentration-response relationships, orthogonal assays, counter-screens or cytotoxicity controls, target validation, *in vivo* replication, and clinically plausible exposure increased confidence (Baell and Holloway, 2010; Magalhães et al., 2021; Bolz et al., 2021).

### 2.4 Rationale for selecting representative metabolites and miRNAs

Plant-derived metabolites and botanical interventions were selected for detailed discussion when they met at least one of four criteria: direct perianal or anorectal evidence; *in vivo* evidence in infected or otherwise mechanistically relevant wounds; a documented link to extracellular-vesicle biology, miRNA regulation, or vesicle-assisted delivery; or a reproducible mechanistic and formulation rationale relevant to local wound repair. Greater interpretive weight was given when more than one evidence tier supported the same biological direction, but reports from the same research programme were not counted as independent replication. Studies that materially changed the interpretation of a clinical or mechanistic question were discussed individually in the relevant section rather than compressed into a citation list. Older, indirect, or methodologically weaker studies were retained when they filled an evidence gap, but they were not given equal narrative weight. Isolated *in vitro* pathway findings without orthogonal or *in vivo* support were retained only as hypothesis-generating evidence.

The miRNAs emphasized in the review had experimental evidence linking extracellular-vesicle transfer to a wound-relevant recipient-cell phenotype or repair outcome and represented complementary biological domains: inflammatory restraint or macrophage reprogramming (miR-146a, miR-223, miR-155), angiogenesis and stromal repair (miR-21, miR-126, miR-132), fibroblast or epithelial recovery (miR-145-5p and related cargos), and pathological anti-angiogenic signaling (miR-24-3p). Expression changes alone were insufficient for mechanistic emphasis; greater weight was given to cargo manipulation, target validation, rescue, or other experiments that tested causality.

## 3 Biology of perianal infectious wound healing

### 3.1 Microbial contamination and biofilm in perianal wounds

The microbiological problem in perianal wounds is not adequately represented by a single planktonic pathogen. Culture-based work identified mixed aerobic and anaerobic organisms in anorectal sepsis, while sequencing of anal fistula-associated sites showed heterogeneous communities with contributions from both intestinal and skin-associated microbiota (Eykyn and Grace, 1986; Yang et al., 2024). Prolonged fistulas can contain organisms with moderate or strong biofilm-forming capacity, providing a plausible mechanism for persistent inflammatory stimulation and impaired tissue repair (Jaiswal et al., 2021).

For this review, the important implication is model validity. Anti-biofilm activity demonstrated against one planktonic laboratory strain cannot by itself establish relevance to a moist, polymicrobial, mechanically disturbed perianal wound. Evidence becomes more informative when it addresses mixed or biofilm-associated infection, tissue injury, host inflammatory responses, and a delivery system capable of maintaining local exposure. These perianal-specific constraints are summarized in Figure 1.

**FIGURE 1:**
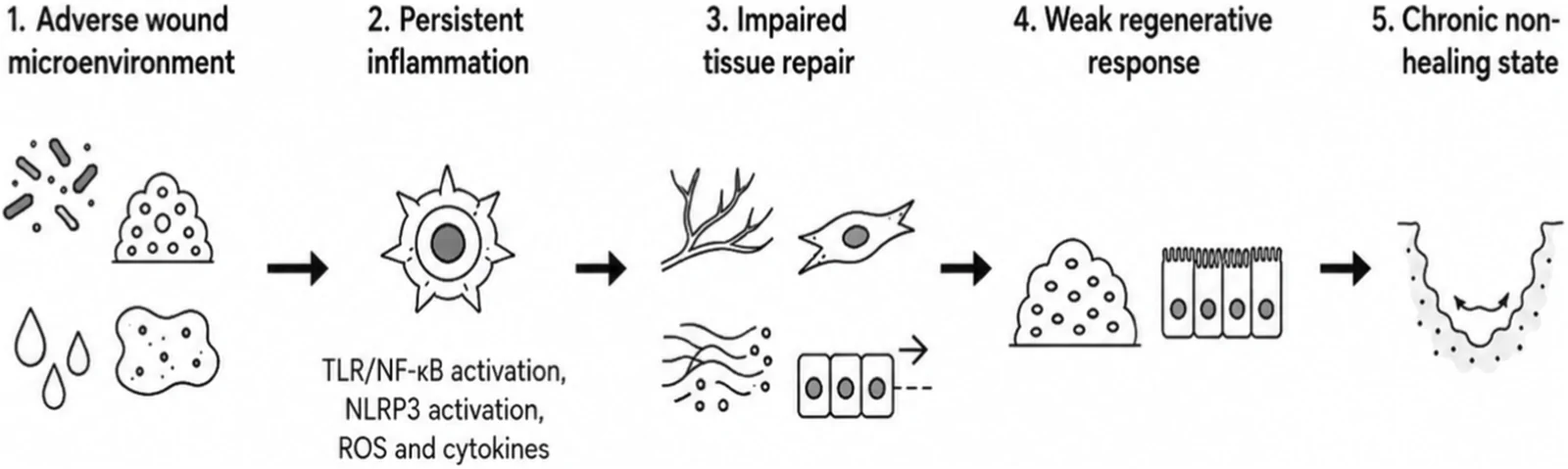
Mechanisms of delayed healing in perianal infectious wounds. This figure outlines a simplified progression of delayed healing. An adverse wound microenvironment with contamination, biofilm, moisture, and tissue debris sustains inflammatory signaling. Persistent inflammation then impairs angiogenesis, fibroblast activity, extracellular matrix remodeling, and epithelial repair, which finally leads to a chronic non-healing state.

### 3.2 Persistent inflammation and impaired regeneration

Persistent microbial and tissue-derived signals can prevent the normal transition from inflammatory defense to repair. Chronic-wound literature shows that sustained cytokine production, oxidative stress, neutrophil proteases, and matrix-degrading enzymes can impair fibroblast migration, endothelial repair, extracellular matrix integrity, and epithelial advancement (Schultz et al., 2011; Landén et al., 2016; Caley et al., 2015). These mechanisms are biologically plausible in perianal wounds because contamination and mechanical irritation are repeatedly renewed.

However, direct molecular profiling of human perianal wound beds remains limited. Findings from diabetic, pressure-ulcer, burn, and dorsal-skin models therefore provide mechanistic analogies rather than proof of perianal pathophysiology. This distinction is central to the evidence hierarchy used throughout the review.

### 3.3 Rationale for targeting intercellular communication

Repair depends on coordinated signaling among macrophages, endothelial cells, fibroblasts, keratinocytes, extracellular matrix components, and microbial stimuli. EVs are relevant because their cargo reflects donor-cell state and can modify recipient-cell behavior (Kalluri and LeBleu, 2020). Experimental wound studies show that vesicle-associated miRNAs can influence angiogenic and inflammatory responses (Lyu et al., 2022), improve diabetic-wound closure when deliberately enriched (Tao et al., 2017), and restore fibroblast function under senescent stress (Su et al., 2023).

Natural products could influence this communication indirectly by changing microbial or inflammatory stress, directly by altering EV-producing cells, or by being incorporated into vesicle-based delivery systems. Yet no human perianal study has shown that a natural product changes wound-fluid EV cargo and that a defined miRNA subsequently causes a healing effect in recipient cells. EV-miRNA signaling is therefore treated here as a testable mechanistic framework, not an established mechanism of perianal therapy.

## 4 Extracellular vesicle-associated miRNA networks

### 4.1 Extracellular vesicles as functional mediators of wound-cell communication

Extracellular vesicles are membrane-delimited particles released by cells and capable of transferring proteins, lipids, nucleic acids, and other molecules. Unless endosomal biogenesis has been demonstrated, the broader term EV is preferable to exosome. This nomenclature is not merely semantic in wound studies, because inflammatory stress, hypoxia, metabolic injury, and microbial exposure can alter both vesicle release and cargo. MISEV2023 therefore places weight on source definition, separation procedures, particle characteristics, positive and negative markers, contaminants, and functional controls rather than on size or a small marker panel alone (Welsh et al., 2024). Natural-product studies require additional care because botanical material, serum-derived material, lipoproteins, hydrogels, and nanoparticles may co-isolate with vesicular fractions and can themselves produce biological activity.

Early clinical experience with locally administered mesenchymal stromal cell EVs emerged from small perianal-fistula cohorts. A five-patient Crohn’s phase I study reported improvement in four patients and complete healing in three after a single local injection (Nazari et al., 2022), and a subsequent 11-patient non-Crohn cohort reported improvement in ten and complete tract resolution in five (Pak et al., 2023). A later Crohn’s phase II study increased the treated population but remained nonrandomized and nonblinded (Hadizadeh et al., 2024). Anatomical relevance is high, but none provides controlled comparative efficacy.

The clinical sequence is better read as an evolving programme than as several independent confirmations. The Crohn’s studies arose from a closely related development network, the non-Crohn cohort remained single-arm, and a later case report combined exosome treatment with salvage surgery (Arslan et al., 2026). Differences in etiology, concomitant procedures, tract anatomy, and natural healing therefore remain alternative explanations for the reported closure signals. The unresolved clinical question is whether a standardized EV product adds benefit beyond contemporary surgical care.

Direct animal models partly address dose, tissue retention, and local biological response. Dose-ranging hUC-MSC-EVs in a complex perianal-fistula rat model and an EV-loaded nanofiber-hydrogel in a Crohn’s perianal-fistula model both improved healing-related endpoints (Lu et al., 2024; Li et al., 2025). The former relies largely on pathway-associated readouts, whereas the latter couples EV activity to an active biomaterial carrier. Their anatomy is closer to the clinical problem than diabetic-skin models, but neither tests natural-product conditioning or a necessary miRNA cargo.

### 4.2 miRNA cargo and recipient cell reprogramming

Assigning a wound effect to an EV-associated miRNA requires more than detecting that miRNA in donor cells or vesicle-enriched material. Selective loading, delivery to the relevant recipient cell, target engagement, and functional dependence are separate experimental questions. Confidence rises when studies combine EV characterization with miRNA gain- or loss-of-function, direct target validation, and rescue; it falls when an expression change is simply placed beside a wound-healing phenotype.

The clearest direct human bridge between a traditional preparation and wound-fluid miRNA biology is the 2025 Wugu Qilin Ointment study. Fifteen patients after low anal-fistula surgery were randomized to Wugu Qilin Ointment, Kangfuxin Solution, or Vaseline dressings; vesicle-enriched material from wound exudate was profiled for miRNAs, and miR-93-3p was subsequently tested in macrophages, where direct mimic/inhibitor experiments and luciferase assays supported EIF4EBP1-related regulation (Zhang et al., 2025). This makes the study more informative than a purely observational expression analysis because it links a human postoperative signal to a functional recipient-cell experiment.

The gap between those two observations is important. Vesicle identity in the clinical samples was supported mainly by ultracentrifugation and morphology rather than a full contemporary MISEV characterization panel, and the macrophage experiments used direct miRNA transfection rather than transfer of patient-derived EVs. No EV-depletion experiment, cargo blockade, or rescue showed that the clinical treatment effect depends on EV-mediated miR-93-3p transfer. Wugu Qilin is therefore direct intersection evidence, but not a closed causal chain. That distinction provides the benchmark for interpreting the better controlled, but anatomically indirect, EV-miRNA wound studies discussed below.

### 4.3 EV-associated miRNAs in inflammatory resolution

In inflammatory wound models, cargo perturbation is more informative than expression profiling alone. Engineered miR-146a EVs reduced IRAK1-related inflammatory signaling and accelerated diabetic-wound closure (Li Q. et al., 2023), while MSC-derived EV miR-223 promoted reparative macrophage polarization through pknox1-related regulation (He et al., 2019); miR-132-enriched EVs produced a similar shift toward reduced inflammation and improved vascular repair (Ge et al., 2023). Defined vesicular cargos can therefore influence the inflammatory-to-reparative transition, although the same cargos have not been shown to change naturally in perianal wound fluid.

Loss-of-function studies provide a complementary test of causality. Suppressing EV-associated miR-155 improved diabetic-wound healing (Gondaliya et al., 2022), and inhibition of diabetes-associated EV miR-24-3p restored angiogenesis through PIK3R3-related signaling (Xu et al., 2020). For perianal wounds, this raises an equally important question to cargo supplementation: whether persistent microbial stimulation maintains harmful vesicular signals that need to be removed rather than replaced.

### 4.4 EV-associated miRNAs in angiogenesis, granulation, and remodeling

Angiogenic EV studies likewise progress from cargo association toward functional perturbation. Cord-blood EV miR-21-3p promoted angiogenic and fibroblast repair (Hu et al., 2018), while M2-macrophage EV miR-21 promoted vascular repair from a different donor-cell context (Lyu et al., 2022). Engineered miR-126-3p EVs delivered in a chitosan dressing went further by improving endothelial proliferation, migration, tube formation, angiogenesis, and diabetic-wound closure (Tao et al., 2017), demonstrating what a defined vascular cargo can achieve when deliberately enriched. These models establish sufficiency more readily than they establish the endogenous cargo composition of a contaminated perianal wound.

Stromal and pathological cargos broaden the same framework. Placenta-MSC EV miR-145-5p restored senescent fibroblast function through CDKN1A-related regulation (Su et al., 2023), whereas diabetes-associated EV miR-24-3p impaired angiogenesis until the miRNA was inhibited (Xu et al., 2020). EV cargo can therefore support or obstruct repair depending on donor-cell state, arguing against a single ‘pro-healing EV signature’ across wound types.

The unresolved perianal question is which of these cargos are present in wound fluid or tissue after fistula or abscess surgery, how they change over time, and whether treatment alters them before clinical healing diverges. Until those measurements are linked to functional transfer experiments, the wound literature identifies candidate cargos rather than a validated perianal EV-miRNA network. Representative EV-associated miRNAs relevant to these processes are listed in Table 1, and the proposed donor-cell–EV–recipient-cell relationships are shown in Figure 2; neither presentation implies that every cargo has been validated in perianal tissue.

**FIGURE 2:**
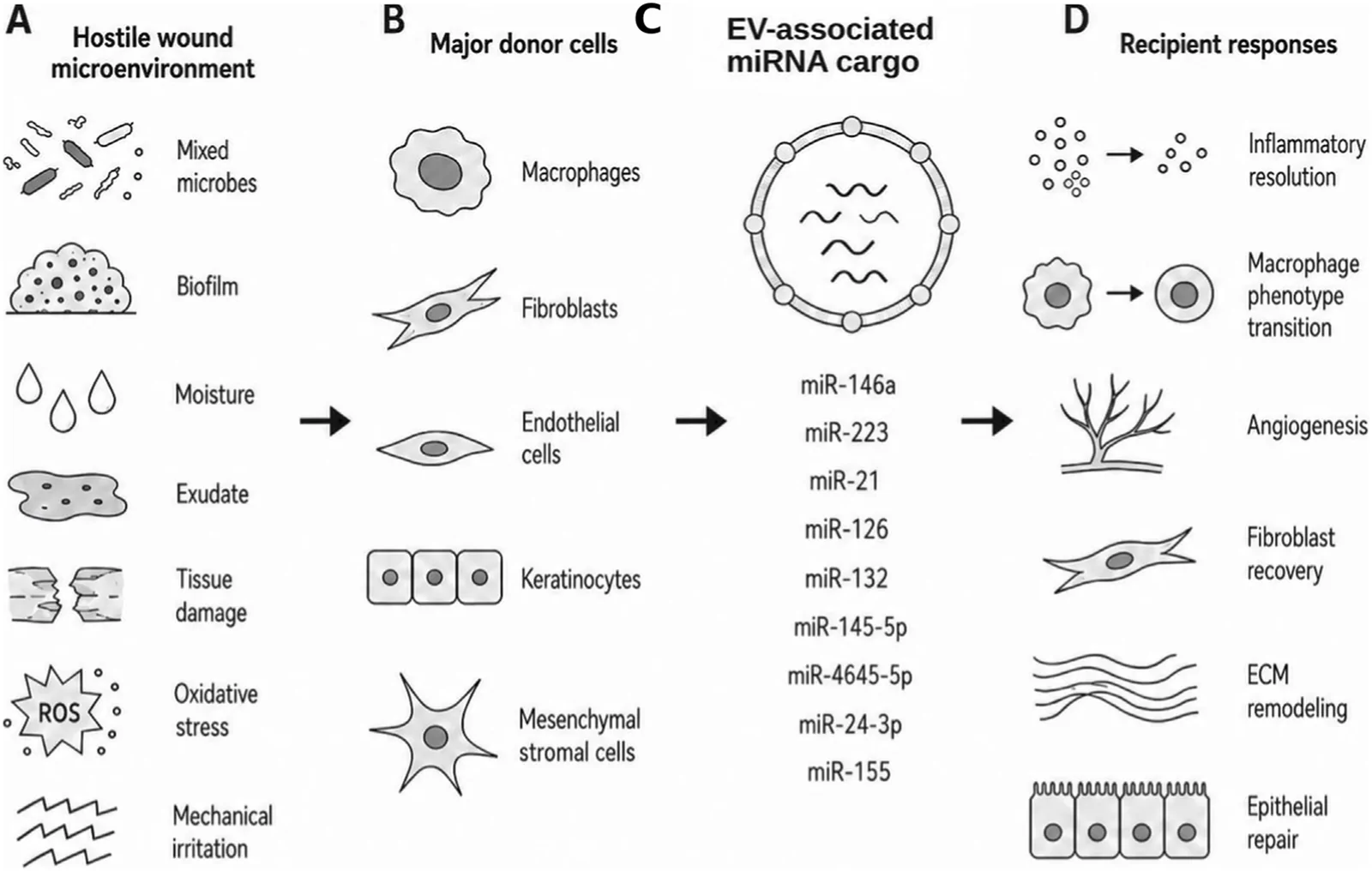
Extracellular vesicle-associated miRNA networks in perianal infectious wound healing. **(A)** Hostile wound microenvironment: mixed microbes, biofilm, moisture, exudate, tissue damage, oxidative stress, and mechanical irritation. **(B)** Major donor cells: macrophages, fibroblasts, endothelial cells, keratinocytes, and mesenchymal stromal cells. **(C)** Candidate EV-associated miRNA cargo: miR-146a, miR-223, miR-21, miR-126, miR-132, miR-145-5p, miR-4645-5p, miR-24-3p, and miR-155. **(D)** Potential recipient responses: inflammatory resolution, macrophage phenotype transition, angiogenesis, fibroblast recovery, extracellular matrix remodeling, and epithelial repair. This is a conceptual network, and not all listed miRNAs or connections have been validated in perianal infectious wounds.

## 5 Plant-derived metabolites and traditional preparations as modulators of infectious wound repair

### 5.1 Anti-inflammatory and immunomodulatory plant-derived metabolites

Inflammation in an infected wound cannot be treated as a process that should simply be suppressed. Neutrophils and macrophages are required for microbial control and debris clearance, while persistent NF-κB activity, inflammasome signaling, oxidative stress, and prolonged inflammatory macrophage states can obstruct the transition to granulation. Natural-product studies are therefore most informative when they distinguish excessive inflammatory activity from necessary host defense and when the tested exposure is pharmacologically interpretable.

Berberine illustrates why pathway claims from cell assays need to be separated from wound-level evidence. Cell studies have linked it to TLR4/MyD88-related macrophage signaling (Gong et al., 2019) and NF-κB/NLRP3-related inflammatory signaling (Chen et al., 2022), but the metabolite is chemically promiscuous and can interfere with some assay formats; these observations therefore support pathway hypotheses more readily than target specificity. Greater translational weight comes from concentration-response biofilm work combined with a 200 μg/mL thermosensitive liposome gel in S. aureus-infected mouse wounds, where formulation-level antibacterial and repair effects were demonstrated (Li S. et al., 2023). Even there, the result concerns an infected-skin formulation rather than a defined molecular target or perianal efficacy.

Baicalin and baicalein have been studied in different experimental settings. A pressure-ulcer study paired keratinocyte testing with high-dose topical baicalin *in vivo* (Kim et al., 2023), whereas baicalein anti-biofilm work used HPLC-confirmed material, concentration-response testing, antibiotic comparators, and several orthogonal microbial assays (Chen et al., 2016). Those controls strengthen the underlying phenotypes, but the pressure-ulcer model is noninfected and the biofilm study lacks host tissue. The evidence is therefore useful for defining plausible activities, not for claiming perianal anti-infective efficacy.

Curcumin creates a different problem because broad *in vitro* target claims are especially vulnerable to assay promiscuity (Baell and Holloway, 2010; Bolz et al., 2021). Li D. et al. (2023) avoided relying on a single target assay by loading curcumin into macrophage-derived EVs and testing the construct in high-glucose endothelial cells and diabetic rat wounds. Endothelial migration, angiogenesis, oxidative-stress control, and wound closure improved with Nrf2/ARE-associated changes. The experiment supports an EV-assisted delivery strategy for curcumin, but it does not show that curcumin changes EV-miRNA cargo or that Nrf2/ARE activation is the necessary mediator of the wound effect.

Quercetin and resveratrol are best interpreted in the same exposure- and model-specific way. Topical 0.1% quercetin ointment improved closure and VEGF/TGF-β1-related repair in a dorsal excision model (Gopalakrishnan et al., 2016), while 16 μg/mL quercetin reduced *Pseudomonas aeruginosa* quorum-sensing transcription without measurably inhibiting growth (Ouyang et al., 2016). Resveratrol at 10 μmol/L was associated with M2-like macrophage responses and improved diabetic-wound healing (Ding et al., 2022). For perianal interpretation, the observed wound and microbial phenotypes are more secure than broad target claims; neither metabolite has been shown to produce a defined EV-miRNA mechanism in a perianal wound.

### 5.2 Antimicrobial and anti-biofilm effects

The microbiological problem in a perianal wound is not adequately represented by growth inhibition of a single planktonic laboratory strain. Anal-fistula microbiota can contain both intestinal and skin-associated organisms, and prolonged disease may include strong biofilm-forming isolates (Jaiswal et al., 2021; Yang et al., 2024). An *in vitro* anti-biofilm signal becomes more relevant when it is reproduced across concentrations, separated from nonspecific bactericidal or cytotoxic effects, and carried into a tissue-containing or infected-wound model.

At the microbial level, sub-inhibitory berberine can reduce quorum-sensing-regulated phenotypes and biofilm formation (Aswathanarayan and Vittal, 2018), while a local berberine-liposome gel extends that observation into S. aureus-infected tissue (Li S. et al., 2023). The transition from a microbial assay to an infected wound substantially improves relevance, but the mouse skin environment still lacks the mixed aerobic-anaerobic community, moisture, and tract geometry of a perianal wound.

Orthogonal biofilm work with baicalein and quercetin adds reproducibility without solving that anatomical gap. Baicalein was assessed across concentrations with CFU, crystal-violet staining, microscopy, virulence readouts, and RT-qPCR (Chen et al., 2016), while quercetin altered major *P. aeruginosa* quorum-sensing regulators at a concentration that did not grossly suppress growth (Ouyang et al., 2016). These are useful anti-biofilm or anti-virulence phenotypes, but mixed-species perianal biofilms and host-cell injury remain untested.

The next useful experimental step is therefore not another single-species screen. Mixed-species biofilms, tissue-containing systems, clinically plausible topical exposure, and simultaneous measurement of bacterial burden and host-cell injury would test whether these anti-biofilm phenotypes survive the conditions that make perianal wounds difficult to treat.

### 5.3 Pro-angiogenic and pro-regenerative metabolites and preparations

Once infection and excessive inflammation are controlled, stable repair depends on perfused granulation tissue, fibroblast function, matrix organization, and epithelial closure. Studies of pro-regenerative natural products are therefore most relevant when they extend beyond a single proliferation assay and evaluate formulation, exposure, vascularization, or wound-level outcomes.

Regenerative evidence for salvianolic acid B progresses from fibroblast responses to formulation-level wound repair. Cell studies reported increased migration and type III collagen production (Szwedowicz et al., 2021), and a self-healing hydrogel subsequently added local delivery, antioxidant, angiogenic, and diabetic-wound endpoints (Zhou et al., 2023). The formulation study is more informative for topical therapy because it addresses retention and tissue delivery, but neither model includes active infection or perianal anatomy.

Astragaloside IV is particularly relevant because it links a defined plant-derived metabolite with EV biology. Xiong et al. (2023) preconditioned endothelial progenitor cells with AS-IV, isolated their EVs, and showed improved angiogenesis, collagen deposition, and closure in type I diabetic rat wounds; rapamycin and wortmannin weakened the PI3K/AKT/mTOR-associated effects. A later hydrogel-assisted AS-IV-EV system reduced ferroptosis and mitochondrial injury while increasing VWF and CD31 (Xiong et al., 2024a). These wound-level studies indicate that donor-cell conditioning can change the regenerative activity of the EV output, but they do not by themselves identify which vesicular cargo is necessary *in vivo*. The cargo question is addressed more directly by the miR-21 and miR-126 studies discussed in Section 6.5.

Ginsenoside Rg1 has a different evidence chain. Earlier endothelial-progenitor-cell work showed improved migration, adhesion, proliferation, and VEGF expression (Shi et al., 2009), whereas Huang et al. (2021) later linked Rg1 to a miR-489-3p/Sirt1 axis in diabetic-wound repair with miRNA gain/loss experiments. That work strengthens target-level interpretation but is not EV-mediated. Ginsenoside Rb1 studies should also be kept distinct from whole-ginseng claims: Lee et al. (2016) treated cultured human dermal fibroblasts with isolated Rb1, so the findings support an Rb1-fibroblast effect rather than efficacy of a chemically characterized Panax preparation.

Centella-derived triterpenes further show why ‘more collagen’ is not a universal endpoint. Asiaticoside and madecassoside promoted collagen-related repair in burn models (Wu et al., 2012), whereas asiaticoside reduced excessive collagen signaling in keloid fibroblasts through Smad-related mechanisms (Tang et al., 2011). Early perianal healing requires matrix deposition, but later remodeling requires restraint of stiff, disorganized scar tissue; the desirable direction of a matrix pathway therefore depends on wound stage.

### 5.4 Traditional-preparation and metabolite-based topical therapy in perianal wounds

Direct human studies are now numerous enough that the main issue is no longer whether a traditional preparation has ever been tested, but how convincingly the clinical design separates treatment effects from perioperative care and expectation. Compound Phellodendron Decoction illustrates this progression. An earlier postoperative study reported improvements in pain, edema, exudation, healing time, inflammatory markers, and granulation-related vascularity, but lacked blinding and detailed material authentication (Deng et al., 2022). A later double-blind placebo-controlled trial retained clinically meaningful healing endpoints and added primary-neutrophil experiments, providing a stronger basis for efficacy while still leaving NETosis as a treatment-associated rather than proven necessary mediator (Wang J. et al., 2026). The two reports are best viewed as development within one clinical programme rather than independent replication.

More recent randomized studies extend direct evidence across different postoperative settings. Modified Hu-Lian-Bi-Guan decoction was evaluated in complex anal fistula with time-to-healing and TGF-β/Smad-related tissue readouts (Yang H.-W. et al., 2026), whereas Radix Arnebiae oil was tested after perianal-abscess drainage against active topical comparators and was associated with faster healing and lower pyroptosis-related markers (Zhao et al., 2026). Both studies move beyond biomarker-only observations because clinical wound outcomes were measured, but incomplete blinding, modest sample size, and parallel pathway measurements mean that neither TGF-β/Smad signaling nor pyroptosis can yet be treated as the mechanism that caused the clinical benefit.

Mechanistic depth has also increased in smaller human studies. Calvatia lilacina treatment was coupled to material characterization and single-cell analysis of postoperative anal-fistula tissue (He et al., 2026), while a randomized Cayratia japonica hydrogel study combined clinical healing with transcriptomic, network, and BMP4-focused experiments (Chen et al., 2026). Single-cell and transcriptomic layers generate testable cellular and pathway hypotheses from human tissue, but their evidentiary weight differs from direct perturbation. Cell-cell communication and network analyses identify associations or candidate pathways; they do not establish that a pathway mediates clinical healing.

Older and adjunctive trials help define the boundaries of the clinical evidence without needing equal space in the main narrative. Qiyu oil gauze (Li et al., 2009), Daphne genkwa extract (Yang et al., 2017), and Zibai ointment (Cai E.-W. et al., 2023) each produced postoperative healing signals, but allocation methods, material standardization, or biomarker-only mechanistic interpretation limit causal depth. Staged herbal treatment (Du et al., 2024) and Neibu Huangqi Youhua studies (Xiong et al., 2025) estimate regimen or add-on effects rather than the effect of an isolated formula. Conversely, a 2026 randomized trial of micronized purified flavonoid fraction improved pain and dressing burden without significantly improving 3-month wound healing, complications, or recurrence, illustrating why symptomatic benefit should not be substituted for wound-healing efficacy (Tsai et al., 2026).

The Wugu Qilin study is considered separately in Section 4.2 because its main importance is not simply another healing signal, but the direct human intersection between a traditional preparation and wound-fluid vesicle-enriched miRNA profiling. Its methodological limits also differ from those of conventional clinical trials: the decisive missing experiment is EV-mediated transfer and cargo dependence rather than another pathway association.

Animal studies are most useful where they answer mechanistic questions that the clinical work cannot. Fistula-like or anal-fistula models have been used to examine macrophage-state transitions (Pang et al., 2025), TGF-β/Smad-related stromal repair (Wang H. et al., 2026), and sequential antibacterial-to-regenerative delivery (Xiao et al., 2026), with stronger inference when perturbation or reversal accompanies the wound phenotype. Perturbation and reversal increase mechanistic depth, but formulation and carrier effects still need to be separated from the activity of individual metabolites or pathways.

Anatomical labels remain important when contamination is simulated outside the perianal compartment. Zuoqing granule and related fecal-contaminated dorsal-wound models can test NET formation, bacterial burden, redox stress, or local delivery under repeated contamination, yet they remain Level 3 infected-wound evidence rather than direct perianal models (Fang X. et al., 2026). Such models may be mechanistically informative without being anatomically equivalent to a fistula tract.

## 6 Evidence linking natural products, EVs, and miRNAs in wound healing

### 6.1 Critical synthesis of the evidence chain

Human postoperative trials now provide direct clinical evidence for several traditional preparations in anal-fistula or perianal-abscess wounds, including studies with active or placebo comparators and clinically meaningful healing endpoints. Their methodological quality is uneven: robust blinding and placebo control remain uncommon, botanical or mixed-material standardization is inconsistent, and molecular readouts are often measured in parallel with healing rather than experimentally shown to mediate it.

Human EV treatment occupies a separate evidence strand. Nazari et al. (2022), Pak et al. (2023), and Hadizadeh et al. (2024) show that local MSC-derived EV administration is feasible in complex perianal fistulas and provide closure signals, but the studies are small, uncontrolled or nonrandomized, and the Crohn’s phase I/II reports are not independent external replications. These trials do not include a natural-product intervention or a defined miRNA mediator. Clinical proximity is therefore high, while comparative efficacy and mechanism remain uncertain.

Wugu Qilin narrows the gap between those two strands because a natural-product-based treatment was associated with altered miRNA profiles in vesicle-enriched human wound exudate and miR-93-3p/EIF4EBP1 was then tested in macrophages (Zhang et al., 2025). What is still missing is the experimental bridge itself: rigorously characterized patient-derived EVs were not shown to transfer miR-93-3p into recipient cells, and blockade of EV release or EV cargo was not shown to abolish the treatment effect. The natural product-EV-miRNA intersection is therefore no longer empty, but it is not yet causally closed.

Anatomically direct animal models add information that cannot be obtained from the early clinical series, particularly about dose, tissue retention, and pathway perturbation. A perianal model with only expression changes may be clinically closer but mechanistically weaker than a diabetic-skin experiment with miRNA inhibition and rescue. Conversely, a sophisticated dorsal contaminated-wound model cannot establish efficacy in fistula-tract anatomy.

### 6.2 Evidence from inflammatory wound models

In inflammatory wound models, cargo perturbation is more informative than expression profiling alone. Engineered miR-146a EVs produced wound-level effects together with functional immune-cell readouts (Li Q. et al., 2023), as did MSC-derived EV miR-223 in a separate macrophage-polarization model (He et al., 2019). These experiments give the cargos greater mechanistic value than studies that simply place a changing miRNA beside a healing phenotype.

Loss-of-function designs extend that logic by asking whether detrimental cargo is necessary. Suppressing EV-associated miR-155 improved diabetic-wound healing, and inhibition of diabetes-associated EV miR-24-3p restored angiogenesis through PIK3R3-related signaling (Gondaliya et al., 2022; Xu et al., 2020). Persistent infection may therefore require attention to harmful as well as regenerative vesicular signals.

Natural-product models can reach comparable mechanistic depth without involving EVs. A fistula-like Danggui Buxue Tang model linked treatment to macrophage-state shifts but did not test pathway necessity (Pang et al., 2025), whereas a fecal-contaminated dorsal model of Zuoqing granule combined dose-response effects on NET formation with Nox4 knockdown (Fang X. et al., 2026). The former is anatomically closer to the clinical problem, whereas the latter provides stronger causal testing; neither advantage substitutes for the other.

### 6.3 Evidence from angiogenesis and granulation models

Vascular repair becomes more persuasive when endothelial function is tied to a defined vesicular cargo. Cord-blood EV miR-21-3p promoted angiogenic and fibroblast responses (Hu et al., 2018), while M2-macrophage EV miR-21 promoted angiogenic signaling from a different donor-cell origin (Lyu et al., 2022). Together, these studies show that reparative vascular signals can be exported from several cell types.

miR-126 offers a clearer progression from engineered sufficiency to natural-product conditioning. EVs deliberately enriched with miR-126-3p improved endothelial function and diabetic-wound closure in a chitosan dressing (Tao et al., 2017), while later AS-IV-conditioned EPC-EV work linked the same cargo to PIK3R2/SPRED1-related signaling with miRNA inhibition across endothelial and wound models (Xiong et al., 2024b). The latter is more directly relevant to the present review because the natural-product exposure, donor-cell response, EV output, and cargo perturbation are connected within one programme, although the disease setting remains diabetic rather than perianal.

Local formulation also matters for regenerative natural products. Salvianolic-acid-B hydrogels provide formulation and tissue-repair evidence (Zhou et al., 2023), AS-IV-conditioned EPC-EVs have the strongest EV connection (Xiong et al., 2023; Xiong et al., 2024a), and the ginsenoside-Rg1 miR-489-3p/Sirt1 model provides miRNA causality without EV mediation (Huang et al., 2021). Keeping those distinctions visible prevents different kinds of mechanistic support from being collapsed into a single ‘pro-angiogenic’ category.

### 6.4 Evidence from fibroblast, keratinocyte, and extracellular matrix remodeling studies

Stable stromal repair has similar cargo-level evidence. Placenta-MSC EV miR-145-5p improved the function of senescent fibroblasts through CDKN1A-related regulation (Su et al., 2023), whereas hypoxic BMSC-derived EV miR-4645-5p supported keratinocyte autophagy and diabetic-wound repair (Shi et al., 2024). The former includes stronger target-level manipulation, whereas the latter broadens the recipient-cell scope to epithelial recovery. Neither has yet been tested at a perianal wound edge.

Natural products acting on the same stromal compartment do not necessarily act through EVs. Salvianolic acid B increased fibroblast migration and collagen-related repair (Szwedowicz et al., 2021), ginsenoside Rb1 altered fibroblast proliferation and matrix readouts *in vitro* (Lee et al., 2016), and Centella-derived triterpenes changed TGF-β/Smad-related collagen synthesis in burn and scar models (Wu et al., 2012; Tang et al., 2011). They are useful for defining plausible recipient-cell phenotypes, but they cannot be cited as evidence that natural products regulate those phenotypes through EV-associated miRNAs.

The clinically relevant endpoint is therefore matrix quality across time rather than maximal collagen deposition. Early granulation requires fibroblast recruitment and matrix formation, while later remodeling requires organized collagen and avoidance of fibrotic closure. Perianal studies should measure these phases separately and should not infer a regenerative mechanism from one late collagen stain or one growth-factor measurement.

### 6.5 Evidence from extracellular vesicle and miRNA-focused natural product studies

Three evidence routes now connect natural products with vesicle biology, but they answer different questions. Wugu Qilin provides the most direct human perianal treatment-associated wound-fluid vesicle-enriched miRNA signal; AS-IV studies test whether a defined metabolite can condition mammalian donor cells and alter EV cargo/function; plant or herb-derived vesicle-like particles act as biological materials or delivery systems in their own right. Treating these routes as equivalent would overstate what any one of them proves.

Among donor-conditioning studies, AS-IV provides the deepest connection between a defined plant-derived metabolite, EV output, and miRNA signaling. In high-glucose endothelial injury, EVs from AS-IV-preconditioned EPCs carried miR-21 and were tested with miRNA manipulation, luciferase analysis, and PTEN rescue (Xiong et al., 2022); the same conditioned EV preparation subsequently improved closure, collagen deposition, and angiogenic markers in diabetic rat wounds (Xiong et al., 2023). The cell experiments provide relatively strong cargo-level causality, whereas the wound study establishes efficacy of the conditioned EV preparation without proving that miR-21 is necessary *in vivo*.

The same programme was later extended to miR-126-3p, linking AS-IV-induced EV release and cargo change with PIK3R2/SPRED1-related signaling and miRNA inhibition in endothelial and diabetic-wound models (Xiong et al., 2024b). Studying exposure, donor-cell biology, cargo, recipient response, and wound phenotype within one programme strengthens internal coherence, but it is not independent external replication and none of the experiments uses a perianal infection model.

By contrast, curcumin- and resveratrol-based vesicle systems primarily answer delivery questions. Curcumin-loaded macrophage EVs improved diabetic-wound repair relative to free curcumin (Li D. et al., 2023), while resveratrol-loaded nanoparticles combined with platelet EVs improved wound outcomes in a composite hydrogel (Zhu et al., 2022). Neither design shows that the natural product reprograms endogenous EV-miRNA cargo; the vesicle is a carrier or co-delivery component rather than evidence of donor-cell conditioning.

Plant- and herb-derived vesicle-like particles form a third route. A Salvia miltiorrhiza-Astragalus membranaceus preparation was characterized by lipidomics and small-RNA sequencing and tested in an infected diabetic-wound hydrogel (Dai et al., 2026). Additional wound-delivery examples include grapefruit-derived EVs (Savcı et al., 2021), orange-derived EVs (Gai et al., 2026), mango-ginger-derived nanovesicles (Suresh et al., 2025), and aloe/ginger/neem-derived systems (Miya et al., 2024). The current data establish feasibility of plant-derived nanovesicles, but candidate small RNAs have not been shown to be necessary for the *in vivo* effects and the materials should not be equated with mammalian exosomes.

No perianal study has yet linked a chemically defined natural-product exposure to rigorously characterized EV cargo change, physical transfer of a defined miRNA to the relevant recipient cell, loss-of-function or rescue of that cargo, and a healing outcome in the same anatomically faithful model. Individual studies cover several parts of this sequence, but none spans the full chain.

### 6.6 Relevance to perianal infectious wounds

Perianal infectious wounds should now be viewed as a setting with direct but fragmented evidence rather than as an evidence-free extrapolation. Randomized postoperative studies show that natural-product-based local regimens can influence clinically measured healing, early human EV studies show that local vesicle therapy is feasible, and Wugu Qilin shows that treatment-associated vesicle-enriched miRNA profiling can be performed in human wound exudate. These strands answer different questions and do not yet validate one another.

The most persistent translational gap is model integration. Diabetic, burn, pressure-ulcer, dorsal infected-wound, microbial, and cell systems remain valuable because they permit dose-response experiments, genetic perturbation, rescue, and detailed cargo analysis. They do not reproduce fistula-tract geometry, sphincter-adjacent tissue, mixed anaerobic/aerobic contamination, moisture, defecation-related mechanical stress, or the clinical requirement for drainage and source control. Anatomically direct models provide that context but often have less mechanistic depth.

A study designed to close this gap would combine: an anatomically faithful, contaminated perianal model; a chemically and taxonomically defined intervention; contemporary EV characterization; longitudinal wound-fluid or tissue miRNA profiling; donor- or recipient-side cargo perturbation; rescue; and clinically interpretable endpoints such as durable closure, recurrence, bacterial burden, and tissue quality. Until such a study is performed, the present evidence supports graded hypotheses and specific experimental priorities, not a single established natural product-EV-miRNA mechanism for perianal wound healing.

## 7 Sources and platforms relevant to natural product-EV-miRNA interactions

### 7.1 Host-cell EVs modulated by natural products

The treatment-associated wound-fluid miR-93-3p signal reported after Wugu Qilin Ointment makes donor-cell origin the next unresolved question. Wound exudate contains vesicles released by macrophages, endothelial cells, fibroblasts, keratinocytes and other injured or infiltrating cells, but the Wugu Qilin study did not identify which compartment produced the altered vesicle-enriched miRNA signal. Natural-product regulation of EV biology therefore requires experiments that connect a defined donor-cell response with the vesicles subsequently recovered from the wound.

Macrophage-derived EVs illustrate how donor-cell state can be linked to a recipient-cell phenotype: M2-macrophage vesicles transfer miR-21 to endothelial cells and promote angiogenic signaling in cutaneous wounds (Lyu et al., 2022). Natural products such as berberine, curcumin, and resveratrol can alter macrophage inflammatory states in separate experiments, but those observations cannot be extended to macrophage EV cargo until vesicle release and composition are measured directly after treatment.

The clearest natural-product-conditioning evidence instead comes from endothelial progenitor cells. Across the AS-IV programme, donor-cell pretreatment was linked first to EV-associated miR-21/PTEN signaling (Xiong et al., 2022), then to wound-level activity of conditioned EVs (Xiong et al., 2023), and later to miR-126-3p/PIK3R2/SPRED1-related regulation with cargo inhibition (Xiong et al., 2024b). This coherent progression is stronger than placing unrelated natural-product and miRNA studies side by side, but it remains one research programme and has not yet been reproduced in infected perianal tissue.

Fibroblasts and keratinocytes are less developed as natural-product-conditioned EV donors even though they are directly exposed to the perianal wound surface. Current studies show that EV cargos can rescue senescent fibroblasts or support keratinocyte repair, but the relevant vesicles generally come from stem cells rather than from wound fibroblasts or keratinocytes treated with a botanical intervention. Measuring how defined topical treatments alter EV output from these resident cells would close an important gap between local pharmacology and wound-cell communication.

### 7.2 Plant-derived extracellular vesicle-like nanoparticles

Plant-derived vesicle-like particles bypass mammalian donor-cell conditioning but create a different validation problem: the biological material itself is heterogeneous. These preparations can contain lipids, proteins, metabolites and small RNAs, and their nomenclature should remain distinct from mammalian exosomes unless biogenesis has actually been demonstrated.

Evidence for plant-derived vesicle-like particles has expanded from edible-plant preparations to medicinal-herb systems. Grapefruit-derived EVs (Savcı et al., 2021), orange-derived EVs (Gai et al., 2026), mango-ginger nanovesicles (Suresh et al., 2025), and aloe/ginger/neem-derived systems (Miya et al., 2024) have produced wound-repair phenotypes in cell or diabetic-wound models. The Salvia miltiorrhiza-Astragalus membranaceus system added lipidomics, small-RNA sequencing, and an infected-wound setting (Dai et al., 2026). The increasing analytical depth is useful, but no individual plant-derived RNA has yet been shown to be necessary for the *in vivo* wound effect, and attribution becomes especially difficult in multi-plant vesicle preparations.

For plant-derived vesicle-like particles, reproducibility depends on defining the active material as clearly as the wound phenotype. Particle isolation, size distribution, morphology, source authentication, batch-to-batch composition, cargo profiling and contaminant controls should be reported together. For perianal translation, stability in infected exudate, uptake by wound cells and performance in mixed-species biofilm or fecal-contamination conditions also need direct testing.

### 7.3 Natural product-loaded EVs and EV-inspired nanocarriers

Curcumin-loaded macrophage EVs illustrate vesicle-assisted delivery rather than donor-cell reprogramming: the carrier improved endothelial and diabetic-wound responses relative to free curcumin, but no endogenous EV-miRNA change was demonstrated (Li D. et al., 2023). Resveratrol nanoparticles combined with platelet EVs answer a related co-delivery question (Zhu et al., 2022), whereas berberine liposomes (Li S. et al., 2023) and berberine-curcumin liposomes (Bhatia et al., 2021) are synthetic carriers with infected-wound or antibiofilm activity rather than EV systems. Keeping these platform classes separate prevents a delivery advantage from being misread as evidence of vesicle-mediated signaling.

A perianal formulation should consequently be judged by retention, release kinetics, cargo stability, antimicrobial performance, exudate compatibility, irritation and the ability to reach irregular cavities without obstructing drainage. Demonstrating molecular activity in a standard dorsal wound is insufficient if the carrier cannot tolerate moisture, bacterial enzymes, fecal components and repeated mechanical disruption.

## 8 Delivery strategies for perianal infectious wounds

### 8.1 Local barriers to topical delivery

Topical therapy is attractive after source control because the intervention can be concentrated at the wound bed, but the perianal environment imposes unusually severe delivery constraints. Moisture, stool contamination, exudate, friction, proteases and repeated cleansing can shorten residence time, while fistula tracts and postoperative cavities create irregular surfaces that are not reproduced by a flat dorsal skin defect.

Retention is the first practical problem. Free curcumin, for example, is limited by poor aqueous solubility and local stability, whereas curcumin-loaded macrophage EVs prolonged delivery and improved diabetic-wound repair relative to free drug (Li D. et al., 2023). The result supports a carrier effect, but the diabetic skin model does not show that the same formulation remains stable in fecally contaminated exudate.

Timing is equally important. Immediately after drainage or fistulotomy, bacterial pressure and excessive inflammation need to be controlled without suppressing the host response required for clearance. Once infection is controlled, vascularized granulation, fibroblast recovery, collagen organization and epithelial advancement become more important. A formulation optimized for only one phase may therefore be less useful than a platform whose release profile changes with wound conditions.

### 8.2 Hydrogels and adhesive dressings

Hydrogels are attractive because they can combine soft-tissue conformity, water retention, controlled release and local coverage, but their value depends on what the model actually tests. Adhesion to wet tissue, exudate exchange, release under protease-rich conditions, irritation and resistance to repeated mechanical disturbance are as relevant to perianal use as the molecular cargo itself.

An early attempt to reproduce perianal contamination used a full-thickness dorsal wound repeatedly exposed to *E. coli* and treated with a dracorhodin-perchlorate adhesive hydrogel (Yin et al., 2022). Material characterization and blinded histology strengthened the experiment, but postoperative antibacterial drugs confounded antibacterial attribution and the wound remained anatomically dorsal. Its main value is therefore as a contaminated-wound retention model rather than direct evidence of perianal efficacy.

More anatomically relevant systems have since combined local retention with mechanistic perturbation. A Danshen/Huanglian-loaded gelatin-Bletilla striata gum dressing was tested in an anal-fistula rat model together with si-TGF-β reversal in fibroblasts (Wang H. et al., 2026), while a pH-responsive hydrogel sequentially released berberine and EGCG in a rat anal-fistula model to link early antibacterial activity with later antioxidant and macrophage-regulatory effects (Xiao et al., 2026). Both improve anatomical relevance, although carrier, formulation, and active-metabolite effects remain partly inseparable.

EV-based biomaterials address the same retention problem from a different direction. A nanofiber-hydrogel containing 3 × 10^11 MSC-derived EVs outperformed surgery alone, carrier alone, MSC-hydrogel, and a tissue-graft comparator in a Crohn’s perianal-fistula rat model (Li et al., 2025). The comparator structure is a strength, but the Crohn’s inflammatory context differs from cryptoglandular infection and the experiment does not identify a natural-product input or a necessary miRNA cargo.

AS-IV-conditioned EPC-EV hydrogels extend the delivery concept into donor-cell modulation. The AS-IV programme shows that conditioned EVs can be incorporated into local biomaterials while retaining angiogenic and cytoprotective activity, including reduction of ferroptosis and mitochondrial injury (Xiong et al., 2024a). For perianal translation, the unresolved issues are residence under contamination, reproducible particle dosing, and whether the cargo that drives diabetic-wound benefit remains active in an infected fistula environment.

### 8.3 Nanoparticles and EV-based delivery

Nanoparticles, liposomes, plant-derived vesicle-like nanoparticles, EVs, and hybrid vesicles provide additional delivery routes. They may improve solubility, tissue penetration, cargo protection, and cellular uptake for plant-derived metabolites such as curcumin, berberine, resveratrol, and quercetin, whose direct topical application may be limited by solubility, stability, or rapid clearance.

Liposomes and nanoparticles remain useful comparators because they can improve solubility and residence without introducing a biological vesicle. Berberine liposome gel improved S. aureus-infected wound healing (Li S. et al., 2023), and berberine-curcumin combinatorial liposomes reduced MRSA biofilm formation and intracellular infection (Bhatia et al., 2021). The formulation studies support antibacterial delivery; they do not establish EV biology and should not be pooled with mammalian or plant-derived vesicles.

Mammalian EVs, engineered EVs and plant-derived vesicle-like particles also should not be treated as one platform class. Curcumin-loaded macrophage EVs primarily act as a drug carrier (Li D. et al., 2023), miR-146a-engineered EVs (Li Q. et al., 2023) and miR-132-engineered EVs (Ge et al., 2023) test defined RNA cargos, and DH-EVs are herb-derived particles with complex endogenous cargo (Dai et al., 2026). Similar wound phenotypes across these systems do not imply a shared EV-miRNA mechanism.

### 8.4 EV-miRNA monitoring as a research strategy

The Wugu Qilin study shows that postoperative wound-fluid vesicle-associated miRNA profiling is technically feasible in human perianal wounds, but it also shows why a clinical miRNA panel would be premature. miR-93-3p changed after treatment and had macrophage effects after direct transfection, yet patient-derived EV transfer was not demonstrated. Candidate cargos such as miR-146a, miR-223, miR-21, miR-126, miR-132, miR-145-5p, miR-155 and miR-24-3p should therefore be measured longitudinally with microbial burden, wound stage, treatment exposure and durable healing before any marker is proposed for clinical monitoring.

## 9 Translational rationale for natural product-EV-miRNA strategies

Natural-product or EV-based treatment should remain adjunctive to drainage, anatomical assessment, debridement when required, indicated antimicrobial therapy and sphincter-preserving surgery. The recent perianal trials are most useful when they test local formulations after source control; they do not support replacing source control with a topical pharmacological strategy.

The clinical studies also suggest that translation should be stage-specific rather than mechanism-specific. Compound Phellodendron Decoction, Radix Arnebiae oil and other postoperative regimens have been evaluated during early inflammatory healing, while staged herbal treatment and several biomaterial studies explicitly change the therapeutic emphasis as granulation develops. Future trials should prespecify wound phase, comparator care, formulation exposure and clinically meaningful endpoints such as time to durable closure, recurrence, pain, exudation and re-intervention rather than relying mainly on pathway biomarkers.

Mechanistic sampling can be embedded within those trials without being allowed to substitute for efficacy. Serial wound fluid or tissue could quantify microbial burden, EV concentration and cargo, inflammatory markers and granulation biology, while randomization and appropriate control groups determine whether healing actually improves. A candidate EV-miRNA should be treated as a pharmacodynamic marker only after its change tracks treatment exposure and outcome reproducibly and, where possible, after donor- or recipient-side perturbation establishes biological relevance.

## 10 Critical discussion, limitations, and research priorities

Recent direct human studies change the evidence base from one of near-complete extrapolation to one of clinically proximal but unevenly reliable data. Randomized postoperative studies now show that several traditional or plant-derived local regimens can alter healing-related outcomes, yet sample size, blinding, comparator choice, formulation standardization and mechanistic depth vary substantially between studies. Clinical proximity therefore cannot be used as a substitute for methodological credibility.

The strongest direct ethnopharmacological evidence comes from completed randomized studies with clinically meaningful wound endpoints rather than from protocols or biomarker-only reports. The 2026 placebo-controlled Compound Phellodendron trial improves on earlier work by adding double blinding and primary-neutrophil experiments, whereas modified Hu-Lian-Bi-Guan decoction and Radix Arnebiae oil provide additional randomized clinical signals with different limitations in blinding and causal interpretation. Calvatia lilacina and Cayratia japonica contribute deeper tissue or multi-omic analysis, but their mechanistic layers should not be allowed to outweigh small cohorts or indirect target validation. Older and add-on studies remain useful for completeness, particularly when their limitations are visible rather than hidden by a single evidence label.

Clinical EV studies address feasibility and local treatment rather than natural-product pharmacology. Nazari, Pak and Hadizadeh provide direct perianal feasibility or signal-of-effect data, but their small uncontrolled designs do not establish comparative efficacy, and the Crohn’s phase I/II reports arise from a closely related development programme. Wugu Qilin directly connects a perianal botanical treatment with wound-fluid vesicle-associated miRNA changes, but incomplete EV characterization and direct miRNA transfection in the recipient-cell experiments leave the transfer step unproven. The field therefore has direct intersection evidence without a complete causal chain.

Several recurring biases should shape interpretation of the remaining literature. Positive wound phenotypes are far more common than neutral results, which makes the MPFF trial useful because symptom improvement occurred without a significant 3-month healing advantage. Related research programmes, including the Compound Phellodendron and AS-IV series, should not be counted as independent replication. In animal work, anatomically faithful fistula models should be separated from dorsal fecal-contamination simulations. In plant-derived metabolite studies, high or poorly justified concentrations, incomplete botanical authentication and assay-promiscuous metabolites such as curcumin, quercetin, resveratrol and berberine increase the need for dose-response testing, viability controls, orthogonal assays and target perturbation rather than broad pathway claims.

EV methodology requires the same discipline. Mammalian EV studies should report source definition, separation method, particle distribution, morphology, positive and negative markers, contamination controls and dose in a manner compatible with MISEV2023 principles. Plant-derived vesicle-like particles require separate source authentication and composition control because their biogenesis and cargo differ from mammalian EVs. A miRNA mechanism is most credible when vesicle transfer is demonstrated and loss-of-function, target validation and rescue connect the cargo to the wound phenotype; abundance alone is not sufficient.

The next experiments should integrate the strengths that are currently scattered across separate study types. Human perianal wound fluid can define the clinically relevant EV and miRNA landscape; anatomically faithful contaminated models can test retention, mixed microbial pressure and mechanical stress; chemically defined interventions can establish interpretable exposure; and donor- or recipient-side cargo perturbation can determine whether a proposed miRNA is necessary. Prospective trials should then test standardized products against appropriate controls while collecting mechanistic samples alongside durable clinical outcomes. Until those elements are combined in the same evidence chain, natural-product modulation of EV-miRNA communication remains a specific, testable hypothesis rather than an established mechanism of perianal wound healing. Table 4 maps these priorities to candidate models, causal tests, and translational endpoints.

**TABLE 4:** Experimental models and translational strategies for validating natural product-EV-miRNA mechanisms.

Validation question	Suggested model	Core assays	Causality test	Translational endpoint	References
Does the perianal wound contain harmful biofilm-associated vesicle signals	Clinical wound fluid and tissue from anal fistula or perianal abscess wounds	16S sequencing, biofilm staining, EV particle analysis and small RNA sequencing	Compare EV-depleted and EV-enriched wound fluid effects on macrophages and endothelial cells	Identify miRNA biomarkers associated with delayed healing	Jaiswal et al., 2021; Yang et al., 2024; Welsh et al., 2024
Can natural products reduce inflammatory vesicle cargo	Macrophages exposed to mixed bacterial products or perianal wound fluid	NF-κB, NLRP3, cytokines, macrophage markers and EV-associated miRNA profiling	Use miRNA mimics, inhibitors and EV release blockade	Select metabolites that lower miR-155 or harmful cargos while increasing miR-146a or miR-223	Taganov et al., 2006; He et al., 2019; Li et al., 2023b; Gondaliya et al., 2022
Can natural products improve angiogenic EV function	Endothelial cells treated with EVs from natural product-conditioned donor cells	Migration, tube formation, VEGF, CD31, VWF and hypoxia response markers	Block miR-126, miR-21 or miR-132 in donor cells or recipient cells	Demonstrate restoration of vascularized granulation	Hu et al., 2018; Tao et al., 2017; Lyu et al., 2022; Ge et al., 2023
Can stromal repair be rescued by vesicle-mediated miRNA changes	Fibroblast and keratinocyte cultures under inflammatory or bacterial stress	Fibroblast senescence, collagen I and III, MMPs, TIMPs, keratinocyte migration and autophagy	Inhibit miR-145-5p or miR-4645-5p and perform rescue experiments	Improve matrix organization and epithelial closure	Caley et al., 2015; Su et al., 2023; Shi et al., 2024
Which delivery system fits the perianal environment	Perianal infected wound animal model with moisture and friction simulation	Retention time, release profile, bacterial burden, cytokines, EV stability and histology	Compare free metabolite, hydrogel, nanoparticle and EV-based delivery	Define a stage-adapted topical formulation	Yin et al., 2022; Lu et al., 2024; Li et al., 2025; Li et al., 2023c; Xiong et al., 2024a
Can the approach be translated clinically	Controlled perianal fistula trial or prospective postoperative anal-fistula wound cohort with etiologic stratification (Crohn vs. non-Crohn/cryptoglandular) and an ethnopharmacological intervention or EV-based intervention	Healing time, pain, exudate, recurrence, MRI/tract closure where appropriate, wound-fluid EV miRNAs, microbial context and safety monitoring	Compare against an appropriate control and test whether EV/miRNA change mediates, rather than merely accompanies, treatment response	Move from early EV feasibility signals to controlled efficacy and natural-product/EV-miRNA mechanistic validation	Nazari et al., 2022; Pak et al., 2023; Hadizadeh et al., 2024; Kang et al., 2022; Qu et al., 2022

## 11 Conclusion

Natural products, extracellular vesicles, and miRNA signaling now intersect at several levels relevant to perianal wound repair. Multiple human ethnopharmacological studies provide direct postoperative efficacy or biomarker data, early human perianal EV studies provide safety/feasibility and closure signals, and Wugu Qilin supplies a direct human treatment-associated wound-fluid vesicle/miRNA bridge. However, no clinical study demonstrates that a natural product improves perianal infectious wound healing through EV-dependent transfer of a defined miRNA, and no study closes the full causal chain from material exposure to EV cargo change, recipient-cell target engagement, and healing outcome.

Current evidence supports a graded interpretation rather than a single mechanistic conclusion. Chemically defined plant-derived metabolites, adequately characterized botanical preparations, and EV-based systems can provide mechanistic and delivery hypotheses, but their relevance depends on model fidelity, exposure, material characterization, appropriate controls, EV quality, and causal validation. Widely studied plant-derived metabolites with potential assay-interference behavior require particular caution when mechanistic claims rest mainly on *in vitro* assays.

A credible translational path begins with source control and clinically derived perianal samples, followed by mixed-biofilm models, rigorous EV-miRNA perturbation, and stage-adapted local formulations tested under realistic moisture, contamination, and mechanical conditions. This evidence-first approach can determine whether modulation of EV-miRNA communication by plant-derived metabolites or botanical preparations becomes a reproducible adjunct to perianal wound care or remains a biologically plausible but unproven concept.
